# Reversing Hydrogen‐Related Loss in α‐Ta Thin Films for Quantum Device Fabrication

**DOI:** 10.1002/advs.202509244

**Published:** 2025-08-11

**Authors:** D. P. Lozano, Massimo Mongillo, Bart Raes, Yann Canvel, Shana Massar, A. M. Vadiraj, Tsvetan Ivanov, Rohith Acharya, Jacques Van Damme, Joris Van de Vondel, Danny Wan, Anton Potočnik, K. De Greve

**Affiliations:** ^1^ Imec Kapeldreef 75 Leuven B‐3001 Belgium; ^2^ Department of Physics and Astronomy KU Leuven Leuven B‐3001 Belgium; ^3^ Department of Electrical Engineering (ESAT) KU Leuven Leuven B‐3001 Belgium

**Keywords:** high‐Q resonators, microwave loss, quantum computing, superconducting qubits, tantalum

## Abstract

α‐Tantalum (α‐Ta) is an emerging material for superconducting qubit fabrication due to the low microwave loss of its stable native oxide. However, hydrogen absorption during fabrication, particularly when removing the native oxide, can degrade performance by increasing microwave loss. This work demonstrates that hydrogen can enter α‐Ta thin films when exposed to 10 vol% hydrofluoric acid for 3 min or longer, leading to an increase in power‐independent ohmic loss in high‐Q resonators at millikelvin temperatures. It is further shown that annealing at 500 °C in ultra‐high vacuum (10^−8^ Torr) for 1 h fully removes hydrogen and restores the resonators’ intrinsic quality factors to ≈4 million at the single‐photon level. These findings identify a previously unreported loss mechanism in α‐Ta and offer a pathway to reverse hydrogen‐induced degradation in quantum devices based on α‐Ta and, by extension also Nb, enabling more robust fabrication processes for superconducting qubits.

## Graphical abstract

α‐Tantalum is one of the best materials for superconducting quantum computers because of its stable, low microwave‐loss oxide. However, excessive amounts of hydrogen can enter the material during fabrication. This study shows that hydrogen degrades quantum device performance, while annealing the device in ultra‐high vacuum removes hydrogen and fully restores its functionality.

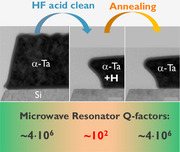

## 1 Introduction

Superconducting qubits have garnered significant attention in quantum information technologies due to their scalability and high‐gate fidelity.^[^ ^1^ ^]^ Despite considerable advancements in qubit performance over the past two decades, further improvements in coherence times and fidelity are essential for realizing practical, large‐scale quantum computers based on superconducting qubits.^[^ ^2^ ^]^ Among the various sources of decoherence, fabrication processes play a critical role, as they can introduce defects and impurities that contribute to microwave loss that in turn limit coherence: examples of such defects include two‐level systems and excess quasiparticles.^[^ ^3^, ^4^, ^5^ ^]^ To achieve high coherence while maintaining compatibility with industrial‐scale methods, qubit fabrication can leverage processing techniques borrowed from the complementary metal‐oxide‐semiconductor (CMOS) industry.^[^ ^6^ ^]^

α‐Tantalum (α‐Ta) has recently emerged as a promising material for superconducting qubits due to its favorable morphological and superconducting properties, including low microwave loss of its stable native oxide.^[^ ^7^, ^8^, ^9^, ^10^, ^11^, ^12^, ^13^ ^]^ These characteristics make α‐Ta a strong alternative to aluminum (Al) and niobium (Nb),^[^ ^14^ ^]^ which are widely used in superconducting qubit fabrication.^[^ ^4^ ^]^ In particular, Ta's native oxide exhibits a more stable and well‐coordinated atomic structure with lower oxygen deficiency compared to Nb oxides, likely resulting in lower magnetic moments density.^[^ ^15^ ^]^ Despite these advantages, α‐Ta—like Nb—has a high hydrogen solubility due to its large interstitial sites in the body‐centered‐cubic (bcc) crystal structure.^[^ ^16^, ^17^, ^18^ ^]^

The presence of diluted hydrogen in Nb metal has been linked to a reduction of high Q‐factors in superconducting radio frequency cavities^[^ ^19^, ^20^, ^21^ ^]^ and, more recently, in planar superconducting Nb resonators^[^ ^22^ ^]^ fabricated using processes similar to those of superconducting qubits.^[^ ^23^, ^24^ ^]^ Hydrogen absorption in Nb—and likely also in α‐Ta films—can occur during various fabrication steps, particularly when the protective native oxide layer, which serves as a hydrogen diffusion barrier,^[^ ^21^, ^22^, ^25^ ^]^ is removed. Hydrogen incorporation can take place during oxide cleaning with fluorine‐based etchants, such as hydrofluoric acid (HF)^[^ ^9^, ^26^ ^]^ or less aggressive buffered oxide etchant (BOE),^[^ ^10^, ^11^, ^27^ ^]^ both commonly used to remove surface oxides and passivate silicon‐air interface. Additionally, hydrogen can enter the metal during acidic or hydrogen‐rich etching processes (e.g. chlorine‐ of fluorine‐based wet^[^ ^7^ ^]^ or dry^[^ ^8^, ^9^, ^14^ ^]^ etching), either through the exposed metal surface during etching or at the sidewalls after etching, where the metal remains unprotected during venting to atmosphere.

While the presence of hydrogen, in the form of niobium hydrides (NbH_x_) precipitates,^[^ ^21^, ^23^ ^]^ and its detrimental impact on microwave loss in high‐Q resonators^[^ ^22^ ^]^ have been established for Nb‐based devices, no evidence of tantalum hydrides (TaH_x_) formation or its potential effect on high‐Q resonators and qubits has been reported or systematically investigated for α‐Ta thin films.

In this study, we investigate the impact of hydrogen absorption in α‐Ta thin films, with a particular focus on its effect on microwave loss relevant to superconducting qubit performance. We show that hydrogen can diffuse into α‐Ta during cleaning with diluted HF, leading to increased power‐independent ohmic loss and suppression of resonance features in superconducting resonators at millikelvin temperatures. Furthermore, we also demonstrate that annealing these films in ultra‐high vacuum (UHV) at 500 °C for 1 h removes hydrogen and fully restores the resonators' quality factors. These findings enable the use of diluted HF for faster and more aggressive surface cleaning and oxide removal, while also providing a technique to reverse the detrimental effects of hydrogen absorption in Ta‐based quantum devices.

## 2 Results and Discussion

### 2.1 Morphology

**Figure 1:**
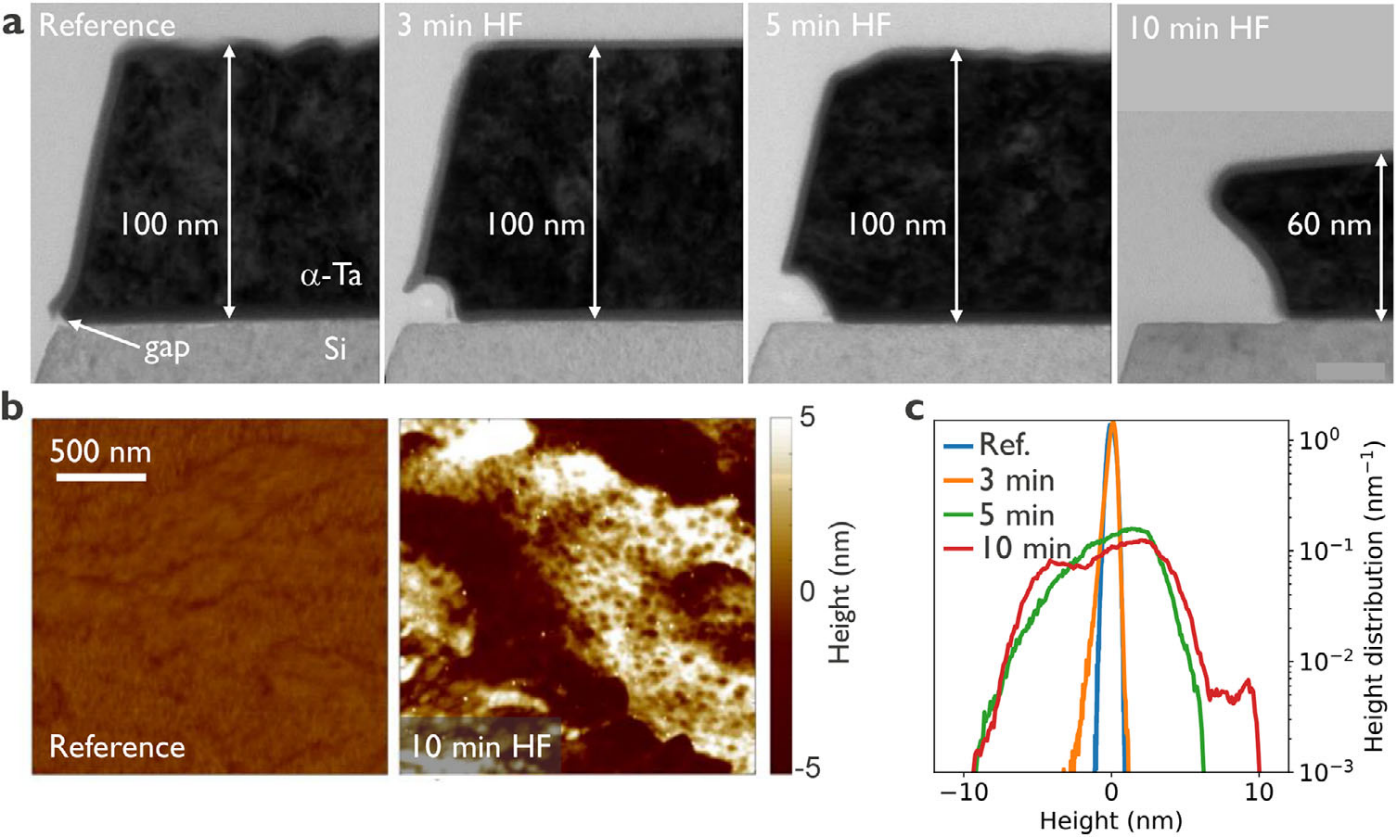
α‐Ta resonators exposed to diluted HF for different durations. a) cross‐STEM micrographs. b) AFM topography of the α‐Ta top surface for reference and 10‐min HF sample. AFM images of all other samples are shown in the Supporting Information. c) Height probability density measured with AFM across the 2 mm × 2 mm area. Root‐mean‐square roughness (R_q_) values for reference, 3 min, 5 min, and 10 min samples are 0.28 nm, 0.38 nm, 2,65 nm, and 3.30 nm, respectively.

Scanning transmission electron microscopy (STEM) analysis reveals that during HF cleaning, α‐Ta etching initiates at the triple point—where the metal‐air, substrate‐air, and substrate‐metal interfaces intersect (**Figure** **1**a). At this location, a small gap forms once SiO_x_ is removed by HF (after ≈1 min),^[^ ^26^ ^]^ exposing the α‐Ta film and making it susceptible to etching. As the duration of HF exposure increases, the etched area gradually extends deeper into the film. The same behavior is observed on several samples, four of which are presented in Figure 1a. STEM micrographs suggest that the triple point serves as the primary pathway for hydrogen infiltration into the film. This is supported by the observation that other regions, specifically the top Ta surface and a large part of the sidewall remain resistant to etching for at least the first ≈5 min of HF exposure. After this time, the HF starts inducing substantial α‐Ta surface modification, indicated by the atomic force microscopy (AFM) measurements (Figure 1b,c). After 10 min HF removes approximately ≈50 nm of tantalum from the sidewall and ≈40 nm from the top surface. The observed difference in etching behavior arises from the varying chemical reactivity of pure α‐Ta and tantalum oxide. Experiments demonstrate that even a brief 1‐min exposure to the HF solution is sufficient to initiate etching of unprotected α‐Ta with an etching rate of ≈15 nm min^−1^. In contrast, tantalum oxide exhibits significantly greater resistance, with an estimated etching rate of 0.4 nm min^−1^, a value that aligns with previously reported findings in the literature^[^ ^28^ ^]^ (see Supporting Information for details).

### 2.2 Hydrogen Content

**Figure 2:**
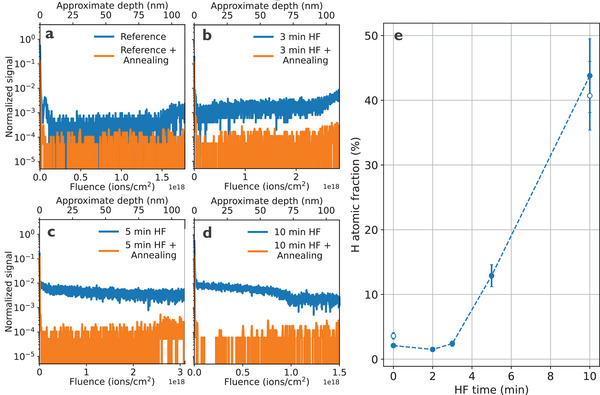
ToF‐SIMS hydrogen signal (normalized counts per second) as a function of fluence or approximate depth for a) reference, b) 3 min, c) 5 min, and d) 10 min HF exposure. Results from samples subjected to a subsequent annealing are plotted in orange color. Silicon signal (not shown here) starts at an approximate depth of 100 nm. e) Energy Recoil Detection Analysis (ERDA) results showing hydrogen atomic fraction as a function of HF exposure time. Empty symbols present additional measurements on a separate set of reference and 10 min HF samples to test reproducibility. Dashed line is a guide to the eye. Error bars represent atomic fraction uncertainties extracting from fitting ERDA spectra. Measurements are performed on the α‐Ta surface hundreds of micrometers away from patterned edges.

The presence of hydrogen diluted in α‐Ta films is first investigated with a time‐of‐flight secondary ion mass spectrometry (ToF‐SIMS) measurements. To avoid hydrogen atoms rapidly discharging from the film into the high vacuum of the sample chamber as soon as the native oxide is sputtered away^[^ ^25^ ^]^ an oxygen (O^2^) sputtering beam is employed, which oxidizes the surface and prevents premature hydrogen desorption during the ToF‐SIMS measurement. The normalized background level of hydrogen is ≈4 × 10^−4^ in the untreated reference α‐Ta sample (**Figure** **2**a). Similar levels are found in samples exposed to HF for 2 min (Supporting Information). The amount of detected hydrogen starts increasing at 3 min and reaches levels of ≈1 × 10^−2^ at 10 min exposure duration (Figure 2b–d). The detection of elevated hydrogen (H^+^) levels throughout the film in the 3‐min sample, combined with the presence of an oxide layer on all other surfaces acting as a strong hydrogen diffusion barrier, suggests that a significant amount of hydrogen is absorbed through the triple point, after which hydrogen spreads relatively quickly throughout the film. This is supported by the fact that the hydrogen diffusion coefficient in Ta is ≈10^−6^ cm^2^ s^−1^ at room temperature,^[^ ^17^, ^18^ ^]^ allowing hydrogen to diffuse across hundreds of micrometers within minutes in a patterned α‐Ta film. In contrast to H^+^, tantalum hydride species (Ta_2_H^−^, TaH_5_^−^, and TaH_x_^−^) are either absent or show levels comparable to the background.

To further confirm the presence of hydrogen in the HF treated α‐Ta samples and identify possible tantalum hydride phases,^[^ ^29^ ^]^ elastic recoil detection analysis (ERDA) is performed to quantitatively determine the atomic fraction of hydrogen in the metal. For the reference, 2 min, and 3 min samples, the hydrogen atomic fraction is comparable, ranging from 3% to 4% (Figure 2e), qualitatively consistent with the ToF‐SIMS data. At these levels, hydrogen is randomly filling predominantly tetragonal interstitial sites in the bcc α‐Ta at room temperature, resulting in a disordered solution of hydrogen in tantalum.^[^ ^29^ ^]^ In the 5 min sample, however, the hydrogen atomic fraction increases to ≈12%, placing it at the boundary between the α‐Ta and α‐Ta + ε phases,^[^ ^29^, ^30^ ^]^ suggesting that ε‐tantalum hydride (ε‐Ta^2^H) might be present. For the 10 min HF sample, the hydrogen atomic fraction reached 40–45%, making δ‐tantalum hydride (δ‐Ta^2^H) also a potential hydride phase present in the film.

Superconducting transition temperature measurements show that α‐Ta films exposed to HF for 10 min do not exhibit superconductivity, whereas films subjected to shorter exposures remain superconducting (see Figure S9, Supporting Information). Furthermore, given that the only known superconducting tantalum hydride forms under extreme pressure (197 GPa),^[^ ^31^ ^]^ we hypothesize that any tantalum hydride precipitates remain metallic at cryogenic temperatures and lead to ohmic loss. These findings suggest that excessive hydrogen incorporation can severely degrade superconducting properties, potentially leading to increased energy loss in superconducting qubits and reduced coherence times.

To eliminate the detrimental effects of incorporated hydrogen, an annealing procedure is employed to fully desorb hydrogen from the α‐Ta film.^[^ ^29^ ^]^ This approach is similar to previously demonstrated methods for hydrogen removal in bulk Nb films.^[^ ^32^ ^]^ The annealing process of HF‐treated samples is carried out at 500 °C for one hour under high vacuum (≈10^−8^ mbar). The removal of hydrogen from the film is confirmed by ToF‐SIMS, where the H^+^ signal in all annealed samples after HF treatment returns to background levels (orange datapoints in Figure 2a–d). The annealing parameters were not optimized, and it is likely that lower values would yield similar results. While prolonged HF exposure strongly affects the metal surface roughness, high‐vacuum annealing does not further modify the surface (see Supporting Information). Notably, annealing restores superconductivity in the 10 min HF‐treated sample, as indicated by the superconducting transition at 4.05 K (see Figure S10, Supporting Information).

In addition to hydrogen, fluorine contamination could also contribute to the degradation of superconducting properties and affect the microwave performance of the resonator. However, ToF‐SIMS spectra of F^−^ (see Figure S6, Supporting Information) show that fluorine levels remain nearly identical across all samples, both after HF treatment and after subsequent annealing. This consistency indicates that fluorine does not play a role in altering the superconducting properties of the film. Instead, the observed changes in our Ta samples can be attributed primarily to hydrogen, highlighting its dominant influence.

### 2.3 Chemical Analysis

**Figure 3:**
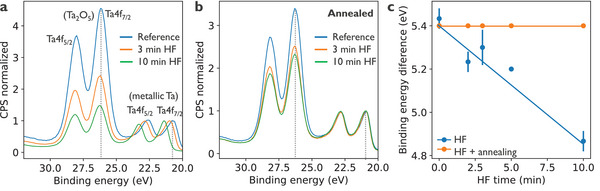
a) Ta binding energy spectrum (normalized counts per second) obtained with surface XPS for selected reference, 3 min HF, and 10 min HF samples. (b) similar to (a) but, with an additional annealing step. Spectra are normalized to the intensity of the metallic Ta4f_7/2_ peak and charge shift is calibrated using C1s peak (see Methods section in Supporting Information). c) binding energy difference between the oxide and the metallic Ta4f_7/2_ peaks indicated with dashed vertical lines for the HF treated samples with and without the high vacuum annealing. Error bars represent the standard deviation calculated from three independent measurements of the HF‐only treated samples.

A more comprehensive understanding of the chemical changes and surface oxidation in α‐Ta films resulting from HF treatment and subsequent high‐vacuum annealing, is obtained through X‐ray photoelectron spectroscopy (XPS) analysis on the α‐Ta top surface, hundreds of micrometers away from the patterned structures. Binding energy spectra show two Ta4f doublets (**Figure** **3**a). The first at 28.0 and 26.2 eV, corresponds to Ta4f_5/2_ and Ta4f_7/2_ peaks of Ta_2_O_5_ and the second doublet at 22.6 and 20.7 eV corresponds to the metallic Ta4f_5/2_ and Ta4f_7/2_ peaks, respectively. The ratio of the Ta_2_O_5_/Ta peak amplitudes decreases with HF exposure duration, indicating that the HF treatment removes excess Ta oxide from the surface (see Figure S8, Supporting Information for more details).

Interestingly, a significant shift of the metallic Ta doublet is observed with increasing HF duration. In the 10‐min HF sample, the Ta4f_5/2_ and Ta4f_7/2_ peaks shift from 22.6 eV to 23.3 eV and from 20.7 eV to 21.4 eV, respectively, compared to the reference sample. This is clearly visible in the binding energy shift, calculated as a binding energy difference between the Ta_2_O_5_ and the metallic Ta4f_7/2_ peaks (Figure 3c). To confirm that this shift is not a measurement artifact, the measurements are repeated on two additional sets of samples, which consistently show the same trend (see Supporting Information). The observed binding energy shift is not related to charging effects, as this would result in the opposite effect, where instead of metallic peak, the oxide peak would gradually shifts to higher energies, and the metallic peak would remain unaffected.^[^ ^33^ ^]^ The above observation, together with the ToF‐SIMS data, suggests that the shift in the Ta metallic peaks arises from a hydrogen‐rich chemical environment around Ta atoms, either due to hydrogen diluted in the film or the formation of tantalum hydrides.

Moreover, the fact that only the metallic peaks are affected, while the oxide peaks remain unchanged, strongly suggests that hydrogen is absorbed exclusively within the tantalum metal and not the oxide. This is further supported by XPS data from annealed samples, where the metallic Ta doublet (Figure 3b) or its corresponding binding energy difference (orange points in Figure 3c) show no shift with increased HF exposure time. This indicates that hydrogen desorption fully restores the electronic environment of the tantalum atoms to that of the untreated reference sample. To our knowledge, this is the first reported observation of such a binding energy shift, since no reference for tantalum hydrides or hydrogen‐rich tantalum films are present in well‐known XPS databases.^[^ ^34^ ^]^

### 2.4 Microwave Loss

The impact of hydrogen incorporation in α‐Ta film on microwave loss is evaluated by measuring the intrinsic quality factor (*Q*_i_) of high‐Q resonators in a dilution refrigerator at ≈10 mK temperature (see Methods in Supporting Information for details). Resonators serve as effective proxies for superconducting qubits, as they are sensitive to many of the same microwave loss mechanisms while being easier and faster to fabricate and measure. Each sample contains eight coplanar‐waveguide resonators with resonant frequencies evenly distributed between 4.2 GHz and 7.8 GHz. All resonators share the same geometry, with a central trace width of *w* = 24 µm and a gap of *s* = 12 µm between the trace and the ground plane.

**Figure 4:**
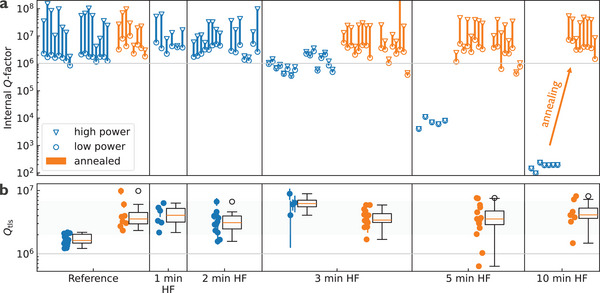
High‐Q resonator measurement results. a) High power (triangle) and low power (circle) internal Q‐factor datapoints are connected with a vertical line for each resonator. Datapoints corresponding to resonators from different samples are separated with a larger gap in horizontal direction than datapoints corresponding to the same sample. Resonators are horizontally ordered according to their frequency. Samples subject to UHV annealing are plotted with the orange color. While time dependence of low power Q_i_ was measured for selected resonators (not shown), their standard deviation is generally lower than resonator‐to‐resonator variation. b) Extracted Q_TLS_ for all measured resonators are presented on a scatter plot grouped by different sample treatments. Error bars indicate the uncertainty in the Q_TLS_ parameter obtained from the nonlinear fit. Datapoints with unreasonable uncertainty, i.e., where the uncertainty exceeded the parameter's value, were excluded from the analysis. Box plot is added to each scatter plot, where the box extends from the lower to upper quartile values of the data, with a line at the median. The whiskers extend from the box and show the range of the data up to 1.5× the inter quartile range (IQR). Flier datapoints beyond that range are considered outliers.

The resonator internal Q‐factor characterization is performed on all resonators across differently processed samples to obtain statistically relevant results. For clarity, only single‐photon level (low‐power) *Q*_i,LP_ and high‐power *Q*_i,HP_ datapoints in the linear regime (up to ≈10^8^ photons) are shown in **Figure** **4**a. A pair of *Q*_i,LP_ ‐ *Q*_i,HP_ datapoints is connected by a vertical line for each resonator. Resonators exposed to HF for 1 and 2 min exhibit approximately twice the median *Q*_i,LP_ (≈4M) compared to the reference sample (*Q*_i,LP_, ≈ 2M), consistent with our previous findings.^[^ ^9^ ^]^ Resonators exposed to HF for 3 min show a notable decrease in *Q*_i_ factors down to *Q*_i,LP_ ≈ 1M, and a negligible power dependence. This correlates with an increased amount of hydrogen absorbed in the 3‐min HF sample (≈4% atomic fraction, see Figure 2e). In samples exposed to HF for 5 and 10 min, resonances show drastically lower *Q*_i_ values of ≈10^4^ and ≈200, respectively. These resonances are particularly challenging to detect and analyze due to the large mismatch between internal and coupling quality factors. Notably, annealing restores internal Q‐factors for all HF‐treated samples, including 5‐ and 10‐min samples (orange points in Figure 4a), indicating that the observed microwave loss is correlated to the amount of hydrogen in α‐Ta films.

To distinguish power‐dependent TLS loss from power‐independent loss, all extracted *Q*_i_ in the linear regime (powers below nonlinear Duffing behavior) as a function of photon number are fitted using a TLS model (Equation S2, Supporting Information) for different samples (Figure 4b). This analysis reveals that TLS loss (1/*Q*_TLS_) is not affected by HF exposure up to 3 min, while the power‐independent loss increases with longer HF exposure affecting both high‐power and low‐power *Q*_i_ (Figure 4a). Furthermore, after annealing, the 3‐min, 5‐min, and 10‐min samples exhibit restored power dependence of *Q*_i_, with TLS loss comparable to that of unannealed 1‐ min and 2‐min HF samples with *Q*_TLS_ ≈ 4M. For the summary of all fitting parameters see Figure S12 (Supporting Information) and subsection Statistical Analysis in Supporting Information for more details.

It is worth noting that the 5‐ min and 10‐min HF‐treated and annealed samples exhibit surface roughness approximately ten times higher than the reference sample (Figure 1b,c; Figure S2, Supporting Information). This increased roughness is expected to raise the participation ratio of the metal‐air interface.^[^ ^4^ ^]^ However, since the *Q*_TLS_ of these samples remains comparable to those with shorter HF exposure and smoother surfaces, this suggests that tantalum oxide at the metal‐air interface is not the dominant loss mechanism in α‐Ta resonators. Furthermore, the silicon‐metal interface is unlikely to be affected by annealing, as the 500 °C annealing temperature is similar to the deposition temperature.^[^ ^9^ ^]^

It is also interesting to note that *Q*_TLS_ of the annealed reference sample is approximately two times higher than that of the non‐annealed reference samples and comparable to 1‐ min and 2‐min HF samples (Figure 4b). This could be a result of modification of the native silicon oxide layer present on the reference sample, reduction of tantalum sub‐oxides (Figure S8, Supporting Information) or removal of microwave TLS loss related to hydrogen^[^ ^35^ ^]^ in the annealed reference sample (Figure 2a). However, further investigation is needed to determine the exact mechanism behind the TLS loss reduction during annealing.

**Figure 5:**
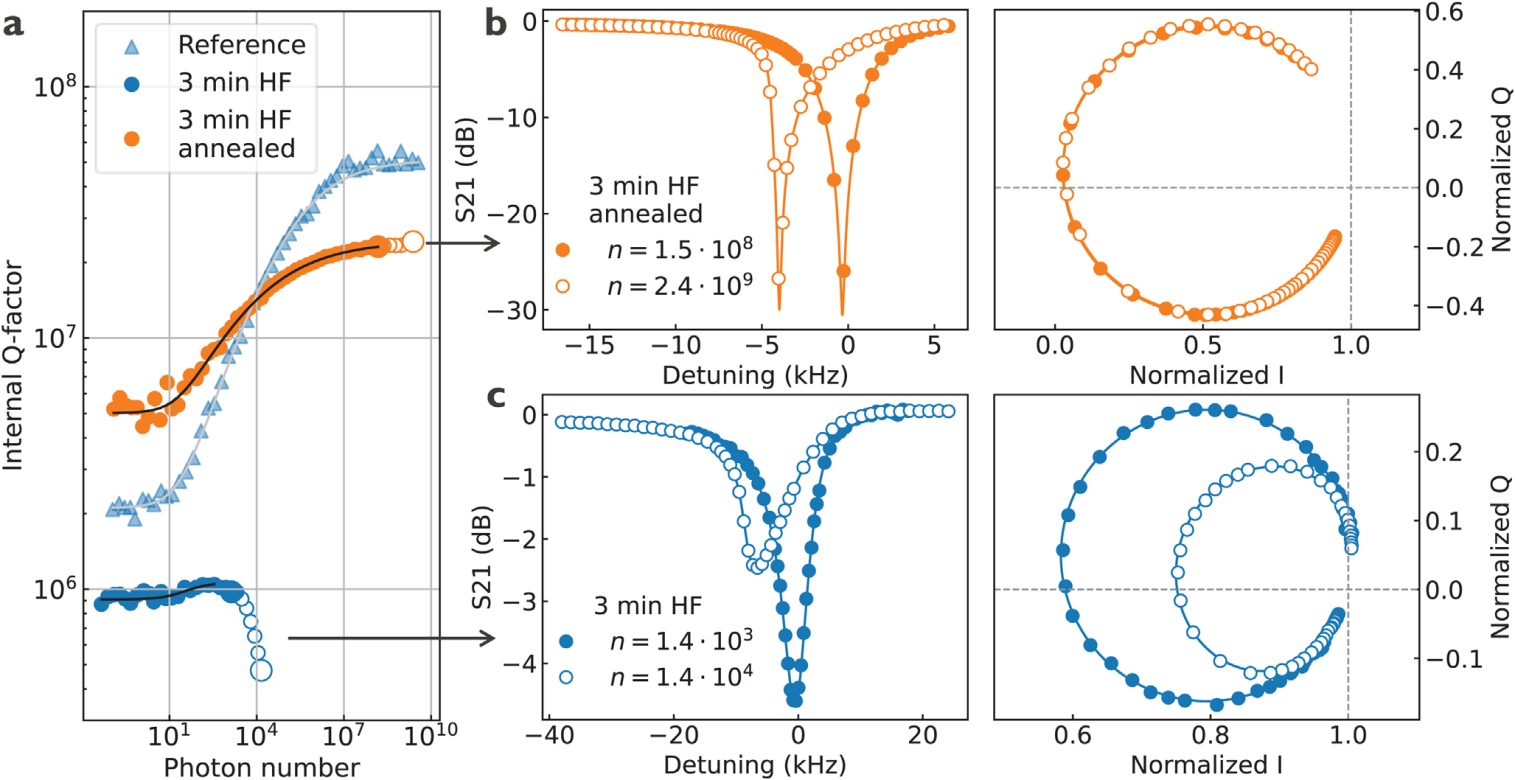
Power dependent internal Q‐factor measurement. a) Power or photon number dependent internal Q‐factor for resonator R1 on samples exposed to different treatments indicated in the legend. Filled markers are obtained by fitting the scattering parameter data to a linear model (Equation S1, Supporting Information), and empty markers are obtained by using a nonlinear model (Equation S8, Supporting Information). b) Scattering parameters as a function of frequency for sample placed in HF for 3 min and received subsequent annealing treatment. The two traces correspond to a linear and nonlinear regime indicated with a photon number in the legend and with large markers in panel (a). c) The same as for panel (b), but on 3 min HF sample that was not thermally treated. Here, non‐circular IQ trace is clearly visible, indicating the presence of nonlinear two‐photon energy loss in high‐Q resonators.

Additional insight into the impact of hydrogen on superconducting circuits can be obtained by analyzing resonator performance at high power levels, beyond the linear regime. At microwave powers above ≈10^8^ photons, superconducting high‐Q resonators typically exhibit Kerr‐type nonlinear behavior.^[^ ^36^, ^37^, ^38^, ^39^, ^40^ ^]^ Interestingly, the 3‐min HF sample displays this nonlinear response at much lower photon numbers, around ≈10^3^. In addition, the onset of nonlinear Kerr‐type frequency shift is accompanied by an increase in microwave loss at higher photon numbers (empty blue circles in **Figure** **5**a). The power dependence of microwave loss can be well modelled with a two‐photon loss mechanism, which is typically associated with quasiparticle heating.^[^ ^36^, ^37^ ^]^ Further supporting this interpretation, the measured scattering parameter S_21_ data in the nonlinear regime exhibit an elliptical shape in the IQ plane (Figure 5c), a consequence of strong power‐dependent loss.^[^ ^36^ ^]^ Fitting a nonlinear model to the measured scattering parameters allows to extract Kerr (*K*_nl_) and two‐photon (γ_nl_) parameters, which are consistent across all resonators on this sample and range between 0.5 and 3 kHz (Figure S11, Supporting Information). For further details on the nonlinear model, see the Nonlinear resonator modeling section in the Supporting Information.

After annealing the 3‐min HF sample, the high‐power behavior of the *Q*_i_ recovers, exhibiting the expected Kerr‐type nonlinearity at expected high photon numbers (≈10^8^ and above). The S_21_ data in the IQ plane retains a fully circular trace, and the extracted Kerr parameter decreases significantly to *K*_nl_ = 5 × 10^−6^ Hz at *n* = 2.4 × 10^9^ (Figure 5b). The same behavior is observed in the reference, the 1‐ and 2‐min HF samples, and in all annealed samples. This value is also consistent with previously reported high‐power characteristics of superconducting high‐Q resonators.^[^ ^38^, ^39^ ^]^

The observed power‐independent loss and nonlinear behavior in α‐Ta samples exposed to HF for 3 min and longer can be explained by ohmic losses due to the formation of non‐superconducting tantalum hydride, or by off‐resonant TLS relaxation‐damping loss and TLS heating—mechanism previously associated with hydrogen in thin Si_3_N_4_ films.^[^ ^35^ ^]^ While the exact origin of the loss remains to be determined, this work reveals a previously unreported hydrogen‐related loss in α‐Ta films, analogous to that observed in Nb resonators.^[^ ^22^ ^]^

## 3 Conclusion

In summary, we demonstrate that prolonged HF exposure of α‐Ta superconducting films introduces a measurable amount of hydrogen into the α‐Ta bcc crystal structure, as confirmed by ToF‐SIMS, ERDA, and XPS. Additionally, extended HF exposure leads to increased power‐independent microwave loss and subsequent suppression of the resonance response in high‐Q resonators, which could be explained by the formation of non‐superconducting tantalum hydride or hydrogen‐induced relaxation dampening TLS loss. Nonlinear resonator analysis further supports this claim, revealing an anomalous two‐photon loss mechanism and increased Kerr nonlinearity in samples exposed to HF for 3 min or longer. However, annealing at 500 °C in UHV effectively removes hydrogen and restores high‐Q performance, bringing *Q*_i_ values back to those of lightly HF‐treated samples. Our results establish a link between hydrogen incorporation and microwave loss in α‐Ta films, offering a clear strategy to reverse hydrogen‐induced degradation in Ta‐ and Nb‐based superconducting quantum devices. This key insight unlocks the potential for more effective etching and cleaning processes in device fabrication, paving the way for enhanced reliability in next‐generation superconducting qubits.

## 4 Experimental Section

Hydrogen incorporation into α‐Ta films was achieved through a hydrofluoric acid (HF) surface oxide removal process.^[^ ^26^ ^]^ Samples prepared with patterned coplanar waveguide resonators on α‐Ta films deposited on silicon wafers, fabricated using the high‐temperature process described in Ref. [9], were submerged in 10 vol% diluted HF for varying durations: 0 min (reference), 1 min, 2 min, 3 min, 5 min, and 10 min. Immediately after the HF treatment, the samples were rinsed in deionized water until the water resistivity in the bath exceeded 12 MΩ.

Hydrogen absorption into the α‐Ta films and further modifications due to the HF treatment were investigated thoroughly on patterned samples with the following material characterization techniques: scanning transmission electron microscopy (STEM), time‐of‐flight secondary ion mass spectrometry (ToF‐SIMS), elastic recoil detection analysis (ERDA), atomic force microscopy (AFM) and X‐ray photoelectron spectroscopy (XPS). The same set of samples was used for ToF‐SIMS, AFM, and XPS measurements and different sets were used for the STEM and ERDA measurements (see Methods section in Supporting Information). In addition to the morphological investigations, high‐Q resonator measurements were performed at 10 mK in a dilution refrigerator designed for high‐coherence superconducting qubit measurements (see Methods section in Supporting Information).^[^ ^9^ ^]^

## Conflict of Interest

The authors declare no conflict of interest.

## Supporting information



Supporting Information

## Data Availability

The data that support the findings of this study are available from the corresponding author upon reasonable request.
