# Supplementary material for: Reversing Hydrogen‐Related Loss in α‐Ta Thin Films for Quantum Device Fabrication
Source: Adv Sci (Weinh). 2025 Aug 11;12(39):e09244. doi: 10.1002/advs.202509244 (PMC12533396; doi:10.1002/advs.202509244)
Supplement: Supplementary file 1 — Supporting Information [file ADVS-12-e09244-s001.docx]

Supporting Information

**Reversing Hydrogen-Related Loss in α-Ta Thin Films for Quantum Device Fabrication**

*Daniel P. Lozano,* Massimo Mongillo, Bart Raes, Yann Canvel, Shana Massar, A. M. Vadiraj, Tsvetan Ivanov, Rohith Acharya, Jacques Van Damme, Joris Van de Vondel, Danny Wan, Anton Potočnik,* and Kristiaan De Greve*

**Contents**

**1.** **Methods** 2

**1.1.** **Scanning Transmission Electron Microscopy (STEM)** 2

**1.2.** **Time-of-Flight Secondary Ion Mass Spectrometry (ToF-SIMS)** 2

**1.3.** **Atomic Force Microscopy (AFM)** 2

**1.4.** **X-ray Photoelectron Spectroscopy (XPS)** 2

**1.5.** **Elastic Recoil Detection Analysis (ERDA)** 2

**1.6.** **High-Q resonator measurements** 2

**1.7.** **Statistical analysis** 3

**2.** **Ta and TaO_x_ etch rates** 4

**3.** **Surface roughness (AFM)** 5

**4.** **ToF-SIMS characterization** 7

**5.** **XPS surface characterization** 10

**6.** **Superconducting *T*_c_ measurements** 15

**7.** **Nonlinear high-Q resonator modeling** 16

**References** 19

# **Methods**

Multiple sample sets were prepared and characterized in this study to validate the effects of HF treatment on patterned Ta films. Resonator samples that had previously undergone HF treatment and annealing were analyzed using microwave characterization and STEM measurements. Microwave characterization was conducted within one day of the HF treatment, whereas STEM measurements were performed 2–3 months later, after the samples had been exposed to ambient conditions. A separate sample set was used for AFM, XPS, and ToF-SIMS measurements, performed both after HF treatment and after annealing. All measurements were conducted within two months of the HF treatment. Additionally, two more sample sets were analyzed with XPS to confirm that the Ta4f_5/2_ and Ta4f_7/2_ shift was caused by the HF treatment. Finally, a separate sample set was used for ERDA measurements. All HF treatments were performed at different times.

## **Scanning Transmission Electron Microscopy (STEM)**

STEM was performed on samples coated with spin-on carbon (SOC) layer. Lamellae with thickness of <50 nm were cut with focused ion beam (FIB) using Helios 450. For this study, HAADF-STEM was used to investigate metal-air, substrate-air and substrate-metal interfaces.

## **Time-of-Flight Secondary Ion Mass Spectrometry (ToF-SIMS)**

ToF-SIMS measurements were performed using a TOFSIMS NCS instrument from ION-TOF GmbH. Negative ion profiles were measured in a dual beam configuration using a Bi^+^ (15 keV) gun for analysis and a Cs^+^ (500 eV) gun for sputtering, while positive ion profiles were measured in a dual beam configuration using a Bi^+^ (15 keV) gun for analysis and a O_2_^+^ (500 eV) gun for sputtering.

## **Atomic Force Microscopy (AFM)**

The AFM measurements were performed using the system ICON PT equipped with Nanoscope V in a tapping mode configuration. The tip used was OCML-AC160TS. Both Ta and Si surfaces were scanned across 2x2 μm^2^ areas with a resolution of 2 nm/pixel.

## **X-ray Photoelectron Spectroscopy (XPS)**

The XPS measurements were carried out in Angle Integrated mode using a QUANTES instrument from Physical electronics. The measurements were performed using a monochromatized photon beam of 1486.6 eV. A 100 micron-wide spot was used. Charge neutralization was used during this experiment. Sensitivity factors specific to each instrument were used to convert peak areas to atomic concentrations. A charge shift calibration was performed on the C1s peak from adventitious carbon (284.8eV) for all spectra.

## **Elastic Recoil Detection Analysis (ERDA)**

ERDA experiments utilized a primary ion beam of ^35^Cl^4+^ accelerated to 8 MeV by a 2 MV tandem accelerator. The forward recoiled and scattered ions are detected with a Time of Flight – Energy (ToF-E) telescope. The telescope has a length of 755.4 mm and is installed at a forward scattering angle of 40°. The sample tilt is at 15°. Scattered Cl was used for Ta. Recoil signals were used for the other elements. The reported concentrations refer to atomic fractions: at%.

## **High-Q resonator measurements**

Hanger-type $\lambda/4$ coplanar-waveguide resonators were used to study microwave loss in Ta resonators subject to different post-fabrication treatments as described in the main text. Resonators have resonant frequencies equidistantly spread between 4.2 and 7.8 GHz with coupling Q-factors ranging between 0.2 M and 1.8 M. Central trace width is *w* = 24 µm and gap between the trace and the ground plane is *s* = 12 µm.

Subdies with 8 resonators are measured in a dilution refrigerator at ~10 mK using Keysight P5004 vector network analyzer (VNA). Detailed information on the experimental setup can be found in our previous work.^1–3^ Frequency dependent complex scattering parameter *S*_21_ was measured near the resonance frequency for all resonators as a function of applied microwave power (Figure 5**b**). Transmission *S*_21_ scattering parameters are analyzed with an generalized linear resonance model derived for a hanger-type resonator geometry and asymmetric line shapes:^4,5^

| $S_{21}=A e^{i(\omega t_{d}+\phi)} \left( 1-\frac{\delta_{c}}{\delta_{c}+\delta_{i}} \frac{1-i\alpha}{1+2i\tilde{\Delta}} \right),$ | (1) |
| --- | --- |

In this expression $A$ is the amplitude of a line shape, $t_{d}$ is electric delay and $\phi$ is the phase. $\alpha$ is a small line shape asymmetry factor ($\alpha\ll1$). $\delta_{i}=\kappa_{i}/\omega_{r}=1/Q_{i}$ is intrinsic loss, $\delta_{c}=\kappa_{c}/\omega_{r}=1/Q_{c}$ is coupling loss and $\tilde{\Delta}=\frac{\omega-\omega_{r}}{\kappa_{i}+\kappa_{c}}$ is normalized frequency detuning. $Q_{i}$ and$Q_{c}$ are intrinsic and diameter corrected coupling quality factors, respectively, as defined in Ref ^6^.

Intrinsic quality factor shows a characteristic power dependence modelled by the two-level-system loss which is expressed as^3,7^

| $\delta_{i}\left( \bar{n} \right)=\frac{1}{Q_{i}\left( \bar{n} \right)}=\frac{1}{Q_{\mathrm{TLS}}}\frac{\tanh\left( \frac{\hbar\omega_{r}}{2k_{B}T} \right)}{\left( 1+\frac{\bar{n}}{n_{c}} \right)^{\alpha}}+\delta_{0},$ | (2) |
| --- | --- |

where $Q_{\mathrm{TLS}}=1/(F \tan\delta_{\mathrm{TLS}})$, is the two-level-system quality factor. *Q*_TLS_ is a function of effective energy participation ratio $F$ of interfaces and where TLS defects reside and $\tan\delta_{\mathrm{TLS}}$, which is the intrinsic loss tangent for the material containing the TLS. $\delta_{0}$ is the contribution from power independent non-TLS loss, $n_{c}$ is the critical photon number related to the saturation electric field of TLS and $\alpha$ is a phenomenological parameter accounting for geometric effects^8^ and the deviation from the standard TLS model.^9^ Furthermore, $\hbar$ is the Planck constant, $\omega_{r}$ is resonator frequency, $k_{B}$ is Boltzmann constant and $T$ is the temperature, comparable to the base temperature of the dilution refrigerator with $T_{\mathrm{base}}\sim10 \mathrm{mK}$.

$\bar{n}$ is average photon number in the resonator calculated as^10^ $\bar{n}=\frac{2P_{\mathrm{in}}}{\hbar\omega_{r}^{2}}\frac{\delta_{c}}{\left( \delta_{c}+\delta_{i} \right)^{2}}$, where $P_{\mathrm{in}}$ is microwave signal power at the input of the resonator. An effective attenuation of approximately 74 dB between the VNA and the resonator inside the dilution refrigerator was separately estimated using ac-Stark shift and $\chi$-shift measurements with a superconducting transmon qubit.^11^

## **Statistical analysis**

We perform a statistical analysis on the extracted *Q*_TLS_ values from several resonators from differently treated samples (see main text). To compare *Q*_TLS_ values across samples, we combine scatter plots with box plots in Figure 4**b** (plotted on a log scale). In each box plot the box spans the interquartile range (IQR), from the 25% to 75% percentile, horizontal line denotes the median, whiskers extend to the furthest datapoints within 1.5 x IQR and points outside this range are marked as outliers.

In the “Microwave loss” section we claim that the following samples exhibit comparable *Q*_TLS_: (i) annealed reference (ii) 1min HF, (iii) 2min HF, (iv) 3min HF, (v) annealed 3min HF, (vi) annealed 5min HF and (vii) annealed 10min HF. To support this, we applied a one-way ANOVA across these seven groups, which contain 8, 6, 14, 4, 16, 16 and 8 *Q*_TLS_ measurements, respectively (see Figure 4**b**). We emphasize that the sample sizes are small, especially in group (iv) where datapoints were removed due to excessive uncertainty in the nonlinear fit, so this analysis is indicative rather than conclusive.

Given that the log-transformed *Q*_TLS_ distribution appears more normally distributed (Figure 4**b**), we conduct ANOVA on both the raw and log-transformed values using *scipy.stats.f_oneway* python package. Both analyses returned *p*‑values above the 0.05 significance threshold, indicating no statistically significant differences in *Q*_TLS_ among these seven samples (see Table 1), corroborating our claim.

**Table 1**: One-way ANOVA results for Q_TLS_ and log-transformed Q_TLS_ across seven treatment groups specified in the text above.

|  | ***p*-value** | ***F*-statistics** |
| --- | --- | --- |
| *Q*_TLS_ values | 0.11 | 1.82 |
| log(*Q*_TLS_) values | 0.24 | 1.38 |

# **Ta and TaO_x_ etch rates**

The etch rate of tantalum oxide was conducted by timed immersion of Ta resonators samples in 10 vol% HF solution. Using atomic force microscopy (AFM), the Ta film height difference was measured between the bottom of the Si resonator gap and the top of the Ta film before and after dipping the samples in HF. Since HF does not attack Si in the range of concentration and treatment durations used in this study, the height difference corresponds to the amount of film removed by the HF. While individual datapoints can be measured with high accuracy, two separate sample sets were measured to gain a better estimate of measurement uncertainty. The first set was immersed for 2, 3, 5, and 10 minutes, while the second set underwent immersion for 8, 9, 10, and 11 minutes. Figure S1 shows that the film thickness remains unchanged up to approximately 9 minutes, with a noticeable reduction visible for 10-minute duration. The 9-minutes step is considered to completely remove the tantalum oxide (~3.7 nm) and marks the beginning of tantalum metal etching. Therefore, we estimate the etching rate of tantalum oxide in a 10 vol% HF solution to be 3.7 nm/9 min = 0.4 nm/min, and the etching rate of the tantalum film to be approximately 15 nm/min. The measured removal of 35 nm of Ta at 10 min is consistent with STEM results shown in Figure 1**a**.


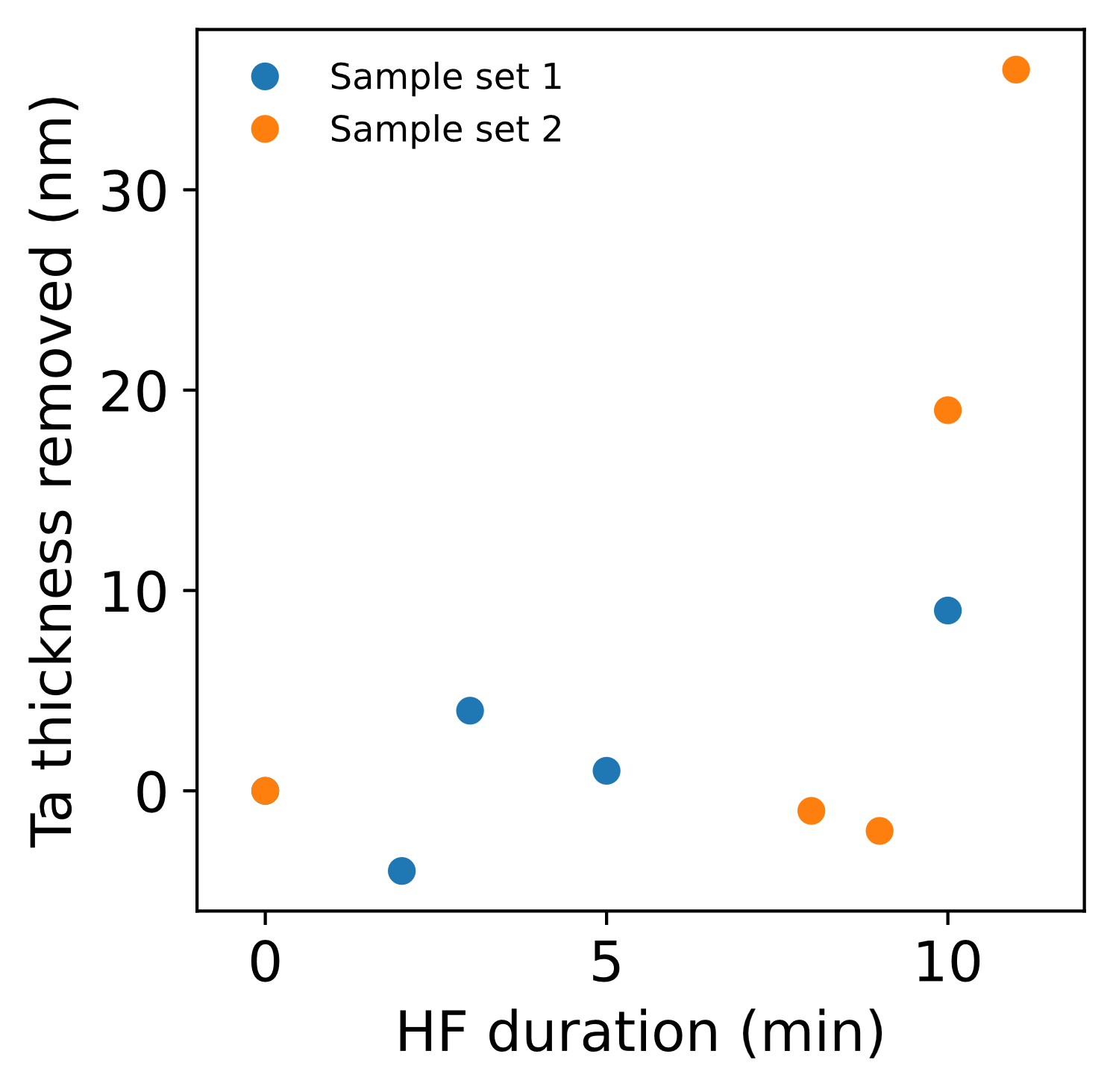


**Figure S1**: Ta film height thickness removed as a function of HF treatment time.

# **Surface roughness (AFM)**

AFM measurements were performed to analyze how the surface topology of Ta resonators evolves with HF treatment duration and annealing. Surface roughness was determined on both Si and Ta surfaces, with measurements taken after HF treatment and again after HF treatment followed by annealing.

The Ta surface height distribution and mean square roughness (*R*_q_) remains consistent across the reference sample and those treated with HF for 2-3 minutes but shows significant increases in the 5- and 10-minute HF samples (Figure S2**a**). These surface characteristics persist after annealing at 500 °C (Figure S2**b**), indicating the thermal treatment does not alter the surface topology. In contrast, the Si surface exhibits no discernible pattern across any of the samples (Figure S2**c**-**d**). This confirms our expectations that neither HF treatment nor annealing affects the Si topology, and that any observed features can be attributed to the Si recess generated during the metal etch step in the samples. All the features discussed above can also be seen in the AFM maps shown in Figure S3**a**-**t**.

**Figure S2**: Height distribution on the **a** the Ta surface after the HF treatment, **b** on the Ta surface after the HF treatment and annealing, **c** on the Si surface after the HF treatment and **d** on the Si surfacer after the HF treatment and the annealing for the reference (blue), 2 min HF (orange), 3 min HF (green), 5 min HF (red) and 10 HF samples d. The inset lists R_q_ values for each of the samples.

**Figure S3**: **a**-**t** AFM scans for the Ta and Si surfaces after the HF treatment and after the HF treatment and annealing.

# **ToF-SIMS characterization**

ToF-SIMS technique is used to detect hydrogen, fluorine and carbon and its distribution in the Ta films. Measurements were taken at two stages: after HF treatment and after subsequent annealing. Figure S4 displays the H^+^ spectra alongside the ^30^Si^+^ signal, which was added to clearly mark the interface between the Ta film and Si substrate. The Ta film thickness of 100 nm was identified at the point where the ^30^Si^+^ signal begins to plateau.

We observe a distinct hydrogen peak at the Ta-Si interface in the 2 min HF and 3 min HF treated samples. In the 5 min and 10 min HF sample, the peak is not visible, likely due to the elevated hydrogen content throughout the film (see Figure S4). While variations in elemental signals can sometimes result from changes in ionization yield at material interfaces (e.g. from Ta to Si),^12^ the weak hydrogen peak in the reference sample suggests a different origin. These observations support the claim that hydrogen initially enters the metal at the Ta-Si-air triple point. An alternative explanation is hydrogen incorporation at the Ta-Si interface from HF during silicon wafer cleaning prior to Ta deposition; however, such hydrogen will likely evaporate during high-temperature Ta deposition process at ~500 °C and, if present, should contribute similarly across all samples.

**Figure S4**: **a**-**e** ToF-SIMS spectra for the H^+^ after the HF treatment (blue) and after annealing (orange). The black line indicates the ^30^Si^+^ signal. Increase of H^+^ signal at the onset of ^30^Si^+^ signal for samples with up to 3-minute exposure to HF could be related to changes in H^+^ ionization yield or hydrogen gradient established due to its entrance through the triple point.

We also monitor the normalized C^-^ (Figure S5) and F^-^ (Figure S6) signals to evaluate whether HF treatments influence the levels of these impurities. All samples exhibit comparable levels of both C^-^ and F^-^, regardless of HF treatment or annealing. This consistency is expected for C, as HF does not effectively remove it, and a thin layer of adventitious carbon inevitably forms upon air exposure, even if surface C is initially removed.

The uniform F signal across samples indicates that F does not influence the superconducting properties of the Ta films, and that the observed shifts in the metallic Ta 4f₇/₂ and Ta 4f₅/₂ XPS peaks are due to hydrogen incorporation rather than the presence of F.

**Figure S5**: a-e ToF-SIMS spectra for the C^-^ after the HF treatment (blue) and after annealing (orange). The black line indicates the ^30^Si^+^ signal.

**Figure S6**: **a**-**e** ToF-SIMS spectra for the F^-^ after the HF treatment (blue) and after annealing (orange). The black line indicates the ^30^Si^+^ signal.

# **XPS surface characterization**

XPS measurements were performed to analyze the oxide composition on α-Ta film surface. Three distinct sample sets were examined in this study. In sample sets 1 and 3, measurements were performed only after HF treatment at varying durations, while sample set 2 (presented in Figure 3) underwent two measurement phases: first after HF treatment and second after subsequent annealing. To assess surface variability, three different points were measured on each sample after HF treatment, whereas for sample set 2, only a single point was analyzed post annealing.

The analysis reveals a progressive shift of the Ta4f metallic peaks toward higher binding energies with increasing HF duration, while the Ta4f peaks corresponding to Ta_2_O_5_ remained stable (Figure 3). Consequently, the binding energy separation between the Ta4f_7/2_ peaks of Ta_2_O_5_ and metallic Ta decreases, as summarized in Figure S7. This shift is reversible, disappearing after 1-hour long UHV annealing at 500°C. Given its reproducibility across three sample sets and reversibility upon annealing, we attribute this behavior to hydrogen absorption and possibly tantalum hydride formation rather than charging effects. The presented data supports the findings in the main text (Figure 3) with additional measured samples, improving statistical reliability.


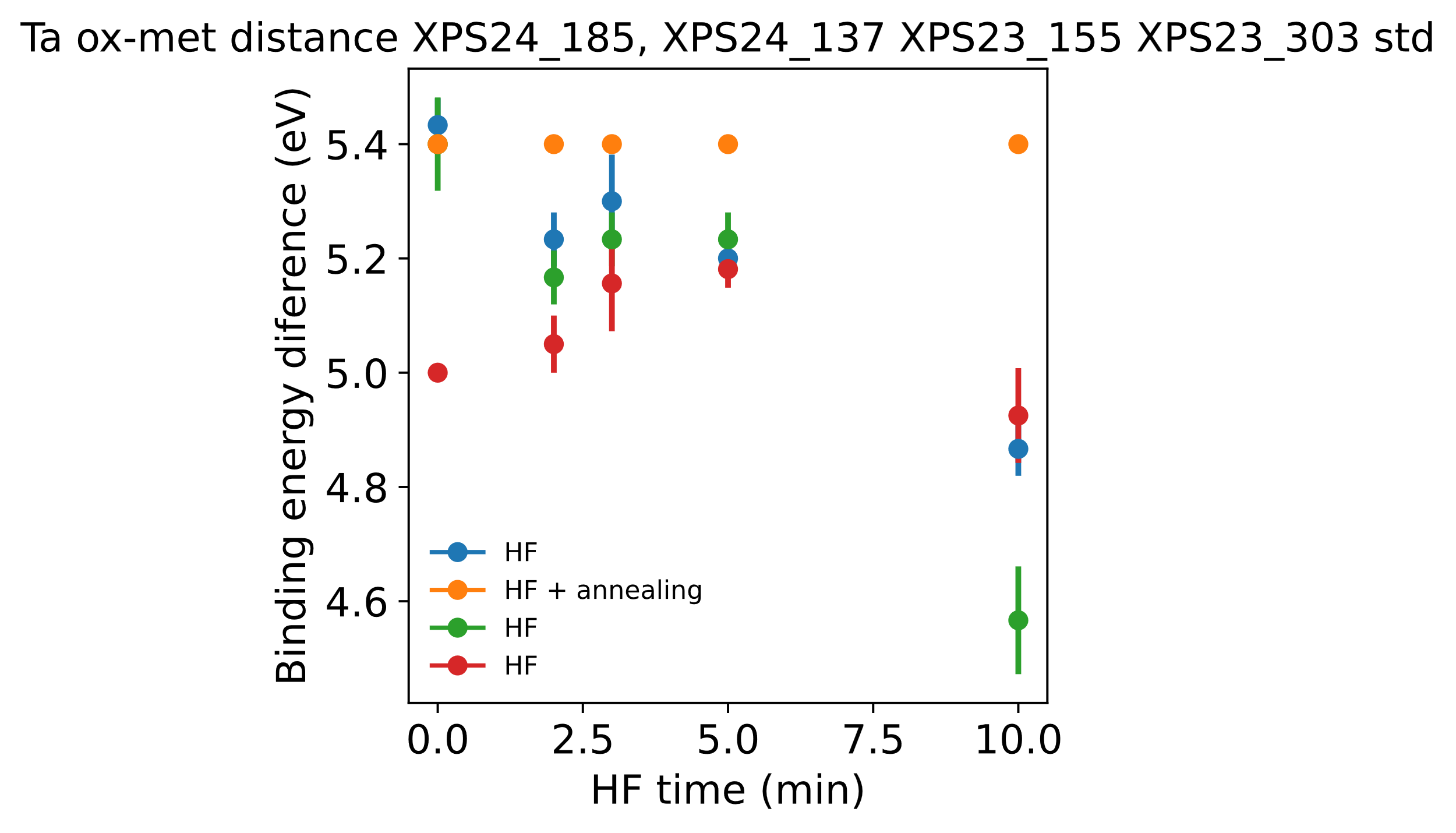


**Figure S7**: Binding energy difference between the Ta4f_7/2_ oxide and metallic peaks for sample set 1 after HF (blue), sample set 1 after HF and annealing (orange), sample set 2 after HF (green) and sample set 3 after HF (red). Markers and error bars represent the mean, and the standard deviation of the binding energy difference extracted from XPS spectra measured on 3 different points in the sample sets 1, 2 and 3 after the HF treatment (9 measurements in total). Sample set two correspond to the data shown in the main text.

To quantitatively determine the amount of tantalum oxide Ta_2_O_5_, tantalum suboxide (TaO_x_) and metallic tantalum components at the surface across sample sets 1, 2, and 3, the Ta4f spectra are fitted with mixed Gaussian-Lorentzian peaks^3,13^ and the relative contributions of individual components are plotted in Figure S8 and summarized in Tables 2-5. While variations in total oxide content exist between sample sets, these differences remain within 15% for any given HF treatment duration. All sample sets exhibited the same trend, which is consistent with our previous results.^3^ Ta_2_O_5_ is the predominant oxide present in all samples. The total amount of tantalum oxides (Ta_2_O_5_ + TaO_x_) reduces from ~85% to ~70% within the first minute of HF exposure and remains largely unchanged with longer exposure times and subsequent annealing. This suggests that ~1-min HF treatment is sufficient to remove the excess tantalum oxide grown during oxygen-plasma-based resist strip,^3^ while any further removal during prolonged HF exposure is offset by native oxide regrowth.

The atomic fractions of tantalum suboxides (TaO_x_) are consistently higher for 2- and 3-minute exposure (blue bars in Figure S8), indicating that this pattern is a genuine phenomenon rather than an artifact of a single measurement set.

It has been hypothesized that surface roughness and pinholes allow the etching solution to access the buried suboxide layer and modify it.^14^ While this is a possible mechanism, the reaction between Ta_2_O_5_ and HF is a complex four-step dissolution process^15^ involving both sub-oxide and pentoxide intermediates that allow for the rearrangement and removal of oxygen atoms from the tantalum oxide structure. Further studies are needed to clarify how acid treatments can modify the suboxide layers in Ta films.

The amount of suboxides decreases significantly in all annealed samples, for instance, from 7% to 3% in the sample exposed to HF for 2 minutes (Figure S8). This suggests that suboxides undergo further oxidation, converting into Ta_2_O_5_. This transformation is expected, as Ta_2_O_5_ is the more thermodynamically stable oxide due to its higher valence state.^16^ Metal suboxides like TaO_x_ typically contain oxygen vacancies caused by deficiency of oxygen atoms in their crystal structure. Upon high-temperature annealing, TaO_x_ undergoes complete oxidation, reducing oxygen vacancies as it converts into Ta_2_O_5_.^17^


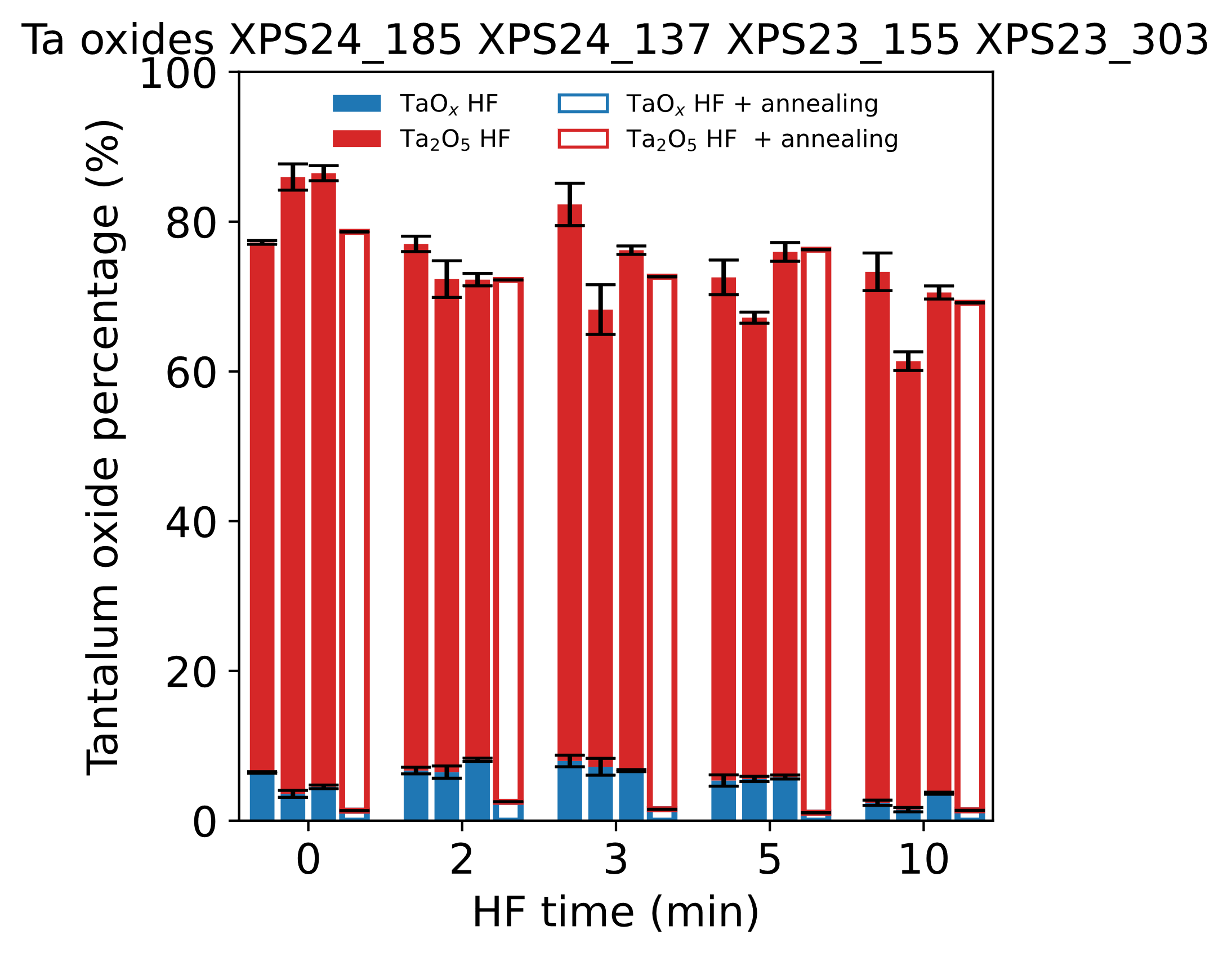


**Figure S8**: Total tantalum oxide determined from fitting XPS spectra for different samples. Different samples are represented with different bars. The amount of suboxide TaO_x_ is indicated by the bottom blue part and tantalum oxide Ta_2_O_5_ amount by the top red part of the bar plot. Annealed samples are indicated by empty bars. Error bars present standard deviation calculated from measurements on three different points for each bar (see Tables 2-5).

**Table 2**: Atomic concentrations extracted at different points on the sample surfaces after fitting the XPS spectrum for the Reference, 2 min HF treated, 3 min HF treated, 5 min HF treated, and 10 min HF treated sample set 1.

| **Reference set 1** | **C (at%)** | **O (at%)** | **Si (at%)** | **Ta met (at%)** | **Ta sub.ox (at%)** | **Ta2O5 (at%)** |
| --- | --- | --- | --- | --- | --- | --- |
| Point 1 | 28.05 | 51.50 | 2.53 | 2.37 | 0.83 | 14.72 |
| Point 2 | 27.61 | 51.16 | 1.66 | 2.68 | 0.86 | 16.02 |
| Point 3 | 24.45 | 53.13 | 2.28 | 2.75 | 0.91 | 16.49 |
| Mean | 26.65 | 51.92 | 2.12 | 2.59 | 0.87 | 15.73 |
| Standard deviation | 1.60 | 0.86 | 0.37 | 0.17 | 0.03 | 0.75 |
| **HF 2 min set 1** |  | | | | | |
| Point 1 | 24.81 | 50.95 | 1.72 | 6.58 | 1.85 | 14.09 |
| Point 2 | 23.12 | 52.85 | 1.01 | 6.16 | 1.81 | 15.06 |
| Point 3 | 23.34 | 52.31 | 1.56 | 6.20 | 1.90 | 14.68 |
| Mean | 23.74 | 52.03 | 1.39 | 6.31 | 1.85 | 14.60 |
| Standard deviation | 0.75 | 0.80 | 0.30 | 0.19 | 0.04 | 0.40 |
| **HF 3 min set 1** |  | | | | | |
| Point 1 | 20.97 | 55.75 | 1.41 | 5.27 | 1.49 | 15.12 |
| Point 2 | 21.00 | 54.79 | 1.27 | 5.44 | 1.53 | 15.97 |
| Point 3 | 21.18 | 55.13 | 0.92 | 5.38 | 1.50 | 15.89 |
| Mean | 21.05 | 55.22 | 1.18 | 5.36 | 1.51 | 15.66 |
| Standard deviation | 0.09 | 0.40 | 0.21 | 0.07 | 0.02 | 0.38 |
| **HF 5 min set 1** |  | | | | | |
| Point 1 | 29.89 | 49.32 | 0.31 | 4.92 | 1.21 | 14.34 |
| Point 2 | 22.91 | 53.95 | 0.73 | 5.39 | 1.29 | 15.74 |
| Mean | 26.17 | 51.58 | 0.48 | 5.15 | 1.25 | 15.02 |
| Standard deviation | 3.49 | 2.32 | 0.21 | 0.24 | 0.04 | 0.70 |
| **HF 10 min set 1** |  | | | | | |
| Point 1 | 23.60 | 52.10 | 1.85 | 7.31 | 0.87 | 14.27 |
| Point 2 | 22.85 | 52.08 | 1.70 | 7.87 | 0.81 | 14.69 |
| Mean | 23.22 | 52.09 | 1.77 | 7.58 | 0.84 | 14.48 |
| Standard deviation | 0.38 | 0.01 | 0.08 | 0.28 | 0.03 | 0.21 |

**Table 3**: Atomic concentrations extracted at different points on the sample surface after fitting the XPS spectrum for the Reference + annealing, 2 min HF treated + annealing, 3 min HF treated + annealing, 5 min HF treated + annealing and 10 min HF treated + annealing sample set 1.

| **Reference + annealing set 1** | **C (at%)** | **O (at%)** | **Si (at%)** | **Cs (at%)** | **Ta met (at%)** | **Ta sub.ox (at%)** | **Ta2O5 (at%)** |
| --- | --- | --- | --- | --- | --- | --- | --- |
| Point 1 | 31.98 | 46.22 | 5.97 | 0.84 | 3.2 | 0.2 | 11.58 |
| **HF 2 min + annealing set 1** |  | | | | | | |
| Point 1 | 29.23 | 47.63 | 3.96 | 0.55 | 5.18 | 0.47 | 12.99 |
| **HF 3 min + annealing set 1** |  | | | | | | |
| Point 1 | 1.19 | 46.09 | 5.08 | 0.57 | 4.67 | 0.26 | 12.14 |
| **HF 5 min + annealing set 1** |  | | | | | | |
| Point 1 | 43.35 | 38.57 | 3.58 | 0.35 | 3.36 | 0.15 | 10.64 |
| **HF 10 min + annealing set 1** |  | | | | | | |
| Point 1 | 31.62 | 45.76 | 5.47 | 0.46 | 5.15 | 0.23 | 11.32 |

**Table 4**: Atomic concentrations extracted at different points on the sample surfaces after fitting the XPS spectrum for the Reference, 2 min HF treated, 3 min HF treated, 5 min HF treated, and 10 min HF treated sample set 2.

| **Reference set 2** | **C (at%)** | **O (at%)** | **Si (at%)** | **Ta met (at%)** | **Ta sub.ox (at%)** | **Ta2O5 (at%)** |
| --- | --- | --- | --- | --- | --- | --- |
| Point 1 | 24.37 | 53.78 | 0.91 | 3.00 | 0.76 | 17.17 |
| Point 2 | 37.45 | 43.27 | 1.74 | 2.42 | 0.61 | 14.51 |
| Point 3 | 24.42 | 53.33 | 0.58 | 3.03 | 0.79 | 17.85 |
| Mean | 28.14 | 49.88 | 0.97 | 2.80 | 0.72 | 16.44 |
| Standard deviation | 6.15 | 4.85 | 0.49 | 0.28 | 0.08 | 1.44 |
| **HF 2 min set 2** |  | | | | | |
| Point 1 | 24.37 | 50.15 | 1.20 | 7.31 | 1.72 | 15.23 |
| Point 2 | 21.24 | 54.25 | 1.25 | 5.47 | 1.29 | 16.50 |
| Point 3 | 22.37 | 51.62 | 1.53 | 7.09 | 1.70 | 15.69 |
| Mean | 22.62 | 51.98 | 1.32 | 6.57 | 1.56 | 15.80 |
| Standard deviation | 1.29 | 1.70 | 0.15 | 0.82 | 0.20 | 0.52 |
| **HF 3 min set 2** |  | | | | | |
| Point 1 | 22.28 | 51.61 | 1.20 | 8.42 | 1.91 | 14.58 |
| Point 2 | 22.46 | 53.73 | 1.08 | 5.99 | 1.40 | 15.34 |
| Point 3 | 21.96 | 51.46 | 0.76 | 8.90 | 2.04 | 14.88 |
| Mean | 22.23 | 52.26 | 0.99 | 7.66 | 1.76 | 14.93 |
| Standard deviation | 0.21 | 1.04 | 0.19 | 1.27 | 0.28 | 0.31 |
| **HF 5 min set 2** |  | | | | | |
| Point 1 | 23.92 | 51.76 | 0.89 | 7.69 | 1.33 | 14.42 |
| Point 2 | 24.79 | 50.59 | 1.27 | 7.88 | 1.38 | 14.08 |
| Point 3 | 24.26 | 51.90 | 0.95 | 7.29 | 1.18 | 14.43 |
| Mean | 24.32 | 51.41 | 1.02 | 7.62 | 1.29 | 14.31 |
| Standard deviation | 0.36 | 0.59 | 0.17 | 0.25 | 0.08 | 0.16 |
| **HF 10 min set 2** |  | | | | | |
| Point 1 | 24.73 | 50.58 | 1.46 | 8.99 | 0.34 | 13.90 |
| Point 2 | 23.88 | 50.48 | 1.03 | 9.98 | 0.48 | 14.15 |
| Point 3 | 24.02 | 52.00 | 0.61 | 8.51 | 0.26 | 14.60 |
| Mean | 24.30 | 50.53 | 1.23 | 9.47 | 0.40 | 14.02 |
| Standard deviation | 0.43 | 0.05 | 0.21 | 0.50 | 0.07 | 0.13 |

**Table 5**. Atomic concentrations extracted at different points on the sample surfaces after fitting the XPS spectrum for the Reference, 2 min HF treated, 3 min HF treated, 5 min HF treated, and 10 min HF treated sample set 3.

| **Reference set 3** | **C (at%)** | **O (at%)** | **Si (at%)** | **Ta met (at%)** | **Ta sub.ox (at%)** | **Ta2O5 (at%)** |
| --- | --- | --- | --- | --- | --- | --- |
| Point 1 | 24.37 | 53.78 | 0.91 | 3.00 | 0.76 | 17.17 |
| Point 2 | 37.45 | 43.27 | 1.74 | 2.42 | 0.61 | 14.51 |
| Point 3 | 24.42 | 53.33 | 0.58 | 3.03 | 0.79 | 17.85 |
| Mean | 28.14 | 49.88 | 0.97 | 2.80 | 0.72 | 16.44 |
| Standard deviation | 6.15 | 4.85 | 0.49 | 0.28 | 0.08 | 1.44 |
| **HF 2 min set 3** |  |  |  |  |  |  |
| Point 1 | 24.37 | 50.15 | 1.20 | 7.31 | 1.72 | 15.23 |
| Point 2 | 21.24 | 54.25 | 1.25 | 5.47 | 1.29 | 16.50 |
| Point 3 | 22.37 | 51.62 | 1.53 | 7.09 | 1.70 | 15.69 |
| Mean | 22.62 | 51.98 | 1.32 | 6.57 | 1.56 | 15.80 |
| Standard deviation | 1.29 | 1.70 | 0.15 | 0.82 | 0.20 | 0.52 |
| **HF 3 min set 3** |  |  |  |  |  |  |
| Point 1 | 22.28 | 51.61 | 1.20 | 8.42 | 1.91 | 14.58 |
| Point 2 | 22.46 | 53.73 | 1.08 | 5.99 | 1.40 | 15.34 |
| Point 3 | 21.96 | 51.46 | 0.76 | 8.90 | 2.04 | 14.88 |
| Mean | 22.23 | 52.26 | 0.99 | 7.66 | 1.76 | 14.93 |
| Standard deviation | 0.21 | 1.04 | 0.19 | 1.27 | 0.28 | 0.31 |
| **HF 5 min set 3** |  |  |  |  |  |  |
| Point 1 | 23.92 | 51.76 | 0.89 | 7.69 | 1.33 | 14.42 |
| Point 2 | 24.79 | 50.59 | 1.27 | 7.88 | 1.38 | 14.08 |
| Point 3 | 24.26 | 51.90 | 0.95 | 7.29 | 1.18 | 14.43 |
| Mean | 24.32 | 51.41 | 1.02 | 7.62 | 1.29 | 14.31 |
| Standard deviation | 0.36 | 0.59 | 0.17 | 0.25 | 0.08 | 0.16 |
| **HF 10 min set 3** |  |  |  |  |  |  |
| Point 1 | 24.73 | 50.58 | 1.46 | 8.99 | 0.34 | 13.90 |
| Point 2 | 23.88 | 50.48 | 1.03 | 9.98 | 0.48 | 14.15 |
| Point 3 | 24.02 | 52.00 | 0.61 | 8.51 | 0.26 | 14.60 |
| Mean | 24.30 | 50.53 | 1.23 | 9.47 | 0.40 | 14.02 |
| Standard deviation | 0.43 | 0.05 | 0.21 | 0.50 | 0.07 | 0.13 |

# **Superconducting *T*_c_ measurements**

Superconducting transition temperature is measured for samples exposed to HF for different durations using a four-probe measurement in an adiabatic demagnetization refrigeration (ADR) cryostat. The reference sample shows the lowest *T*_c_ = 4.20 K, while samples exposed to HF between 2- and 5-minute exhibit somewhat higher values of *T*_c_ ≈ 4.25 K (Figure S9). This increase could be a result of the reduced amount of total Ta oxide on the surface. Surprisingly, the sample that was exposed to HF for 10 minutes shows no superconducting transition down to 0.2 K. This might be due to the considerable amount of hydrogen absorbed or even TaH_x_ formation (close to d phase) in the metal which could render the entire film non-superconducting.


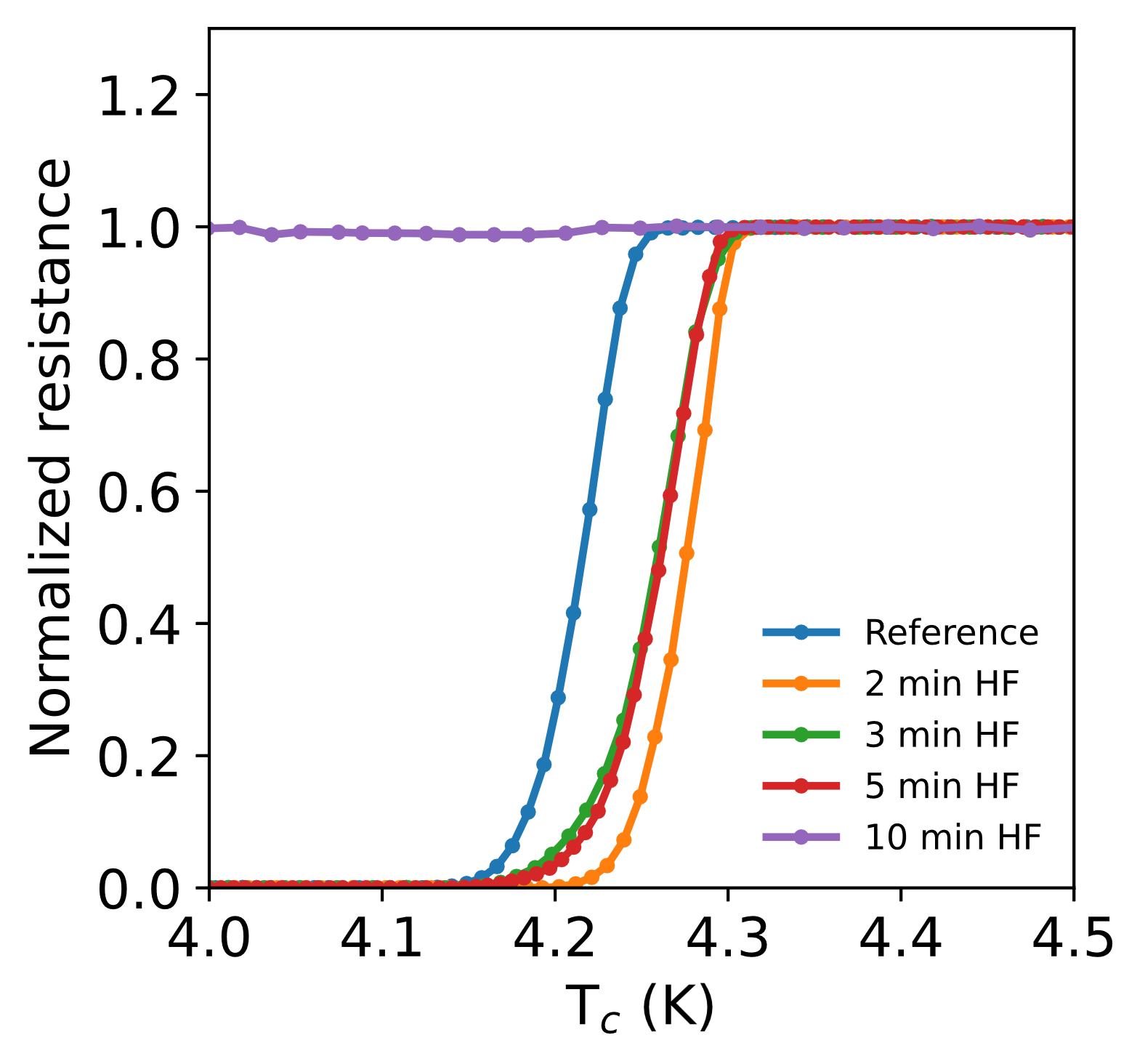


**Figure S9**: Normalized resistance measurements as a function of temperature for Ta samples show superconducting transition at approximately T_c_ = 4.25 K for samples exposed to HF of up to 5 min, and no transition for 10-minute exposed sample down to 0.2 K (not shown).

After annealing the 10-minute HF-treated sample, superconductivity is recovered with *T*_c_ of approximately 4.05 K (Figure S10). Reduced *T*_c_ compared to the reference value (4.20 K) is likely due to the sample being thinner (60 nm) than the reference sample (100 nm) as a result of Ta etching caused by HF treatment.^18^


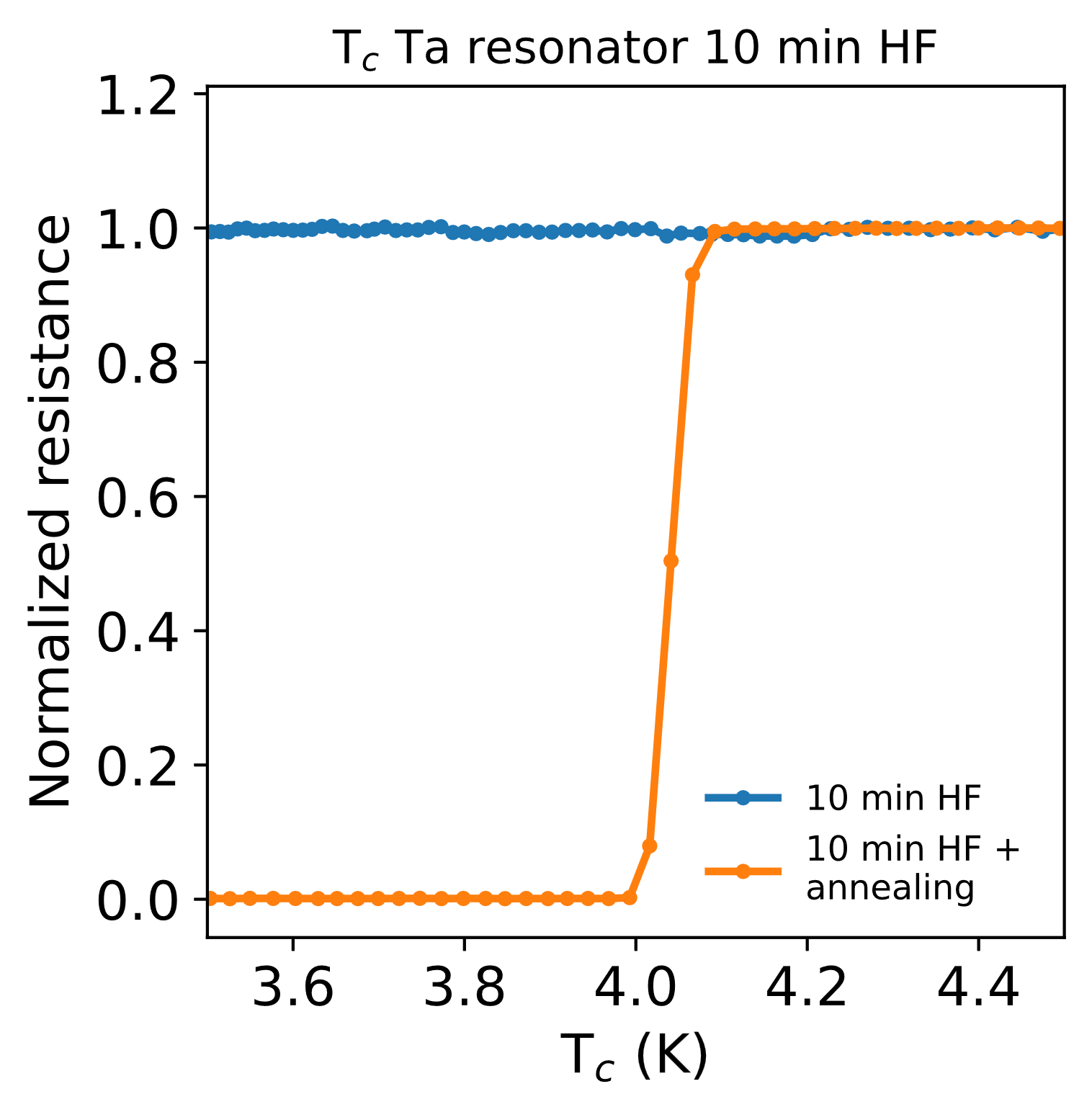


**Figure S10**: Normalized resistance measurements as a function of temperature for Ta samples after 10 min HF (no superconducting transition down to 0.2 K, not shown) and after 10 min HF and annealing showing a superconducting transition at approximately T_c_ = 4.05 K.

# **Nonlinear high-Q resonator modeling**

In this section we describe the nonlinear model and a fitting strategy for measured *S*_21_ scattering parameter as a function of frequency for Ta resonators exposed to diluted HF for 3min (Figure 5). The nonlinear fitting model encompasses both nonlinear kinetic inductance ($L$) contribution as well as nonlinear ohmic loss ($R$) modelled with two-photon absorption contributions^19^

| $L=\Delta L\left( 1+\frac{I^{2}}{I_{c}^{2}} \right), R=\Delta R\left( 1+\frac{I^{2}}{I_{c}^{2}} \right),$ | (3) |
| --- | --- |

where $I$ and $I_{c}$ are current and critical current, respectively. Both contributions arise when microwave current density through a superconducting transmission or a strip line becomes overcritical at its peak near the edges, in which case reduced Cooper pair density and increased quasiparticle density at the edges lead to current dependent kinetic inductance and resistance per unit length.^19,20^

The nonlinear Kerr effect is modelled with the following Hamiltonian,

| $\mathcal{H=\hbar}\omega_{r}a^{\dagger}a+\frac{\hbar}{2}K_{\mathrm{nl}}a^{\dagger}a^{\dagger}aa,$ | (4) |
| --- | --- |

and the two-photon loss is accounted for with the following equation of motion,

| $\dot{a}=-i\omega_{r}a-iK_{\mathrm{nl}}a^{\dagger}aa-\frac{\kappa_{i}+\kappa_{c}}{2}a-\frac{\gamma_{\mathrm{nl}}}{2}a^{\dagger}aa-\sqrt{\frac{\kappa_{c}}{2}}a_{\mathrm{in}}.$ | (5) |
| --- | --- |

In the above two expressions, $\hbar$ is the Planck constant divided by $2\pi$, $\omega_{r}$ is the resonator’s fundamental frequency, $K_{\mathrm{nl}}$ is the nonlinear Kerr parameter, $\gamma_{\mathrm{nl}}$ is the nonlinear two-photon loss parameter, $\kappa_{i}$ is internal loss rate, $\kappa_{c}$ is coupling rate to the feedline, $a^{\dagger}$ and $a$ are creation and annihilation operators obeying bosonic commutation relation $\left[ a,a^{\dagger} \right]=1$ and $a_{\mathrm{in}}$ is incoming drive photon flux.

Assuming coherent drive ($a(t)=ae^{-i\omega t}$) and multiplying both sides of Eq. (5) with its complex conjugate we arrive at the nonlinear equation for inter resonator photon number,

| $\frac{\kappa_{c}}{2}\left\vert a_{\mathrm{in}} \right\vert^{2}=n^{3}\left( K_{\mathrm{nl}}^{2}+\frac{\gamma_{\mathrm{nl}}^{2}}{4} \right)+2n^{2}\left[ \frac{\left( \kappa_{i}+\kappa_{c} \right)\gamma_{\mathrm{nl}}}{4}-\Delta K_{\mathrm{nl}} \right]+n\left[ \frac{\left( \kappa_{i}+\kappa_{c} \right)^{2}}{4}+\Delta^{2} \right],$ | (6) |
| --- | --- |

where $n=a^{\dagger}a$is intra-resonator photon number and $\Delta=\omega-\omega_{r}$ is frequency difference between the drive and the resonator’s fundamental resonant mode.

Eq. (6) can be further simplified by normalizing the equation to^21,22^

| $\frac{1}{2}=\tilde{n}^{3}\left( \xi^{2}+\frac{\eta^{2}}{4} \right)+2\tilde{n}^{2}\left[ \frac{\eta}{4}-\xi\tilde{\Delta} \right]+\tilde{n}\left[ \frac{1}{4}+\tilde{\Delta}^{2} \right],$ | (7) |
| --- | --- |

where

| $\tilde{n}=\frac{n}{\left\vert\tilde{a}_{\mathrm{in}} \right\vert^{2}},$ | $\xi=\frac{\left\vert\tilde{a}_{\mathrm{in}} \right\vert^{2}K_{\mathrm{nl}}}{\kappa_{i}+\kappa_{c}},$ | $\eta=\frac{\left\vert\tilde{a}_{\mathrm{in}} \right\vert^{2}\gamma_{\mathrm{nl}}}{\kappa_{i}+\kappa_{c}},$ | $\tilde{\Delta}=\frac{\Delta}{\kappa_{i}+\kappa_{c}},$ | $\left\vert\tilde{a}_{\mathrm{in}} \right\vert^{2}=\frac{\kappa_{c}}{\left( \kappa_{i}+\kappa_{c} \right)^{2}}\left\vert a_{\mathrm{in}} \right\vert^{2} .$ |
| --- | --- | --- | --- | --- |

When calculating the scattering parameter of a nonlinear resonator, the third order nonlinear Eq. (7) is solved first to obtain $\tilde{n}$ as a function of frequency $\tilde{\Delta}$, which is then used to expand the Lorentzian line shape^[[1]](#footnote-1)^ as follows:

| $S_{21}=A e^{i(\omega t_{d}+\phi)} \left( 1-\frac{\delta_{c}}{\delta_{c}+\delta_{i}} \frac{1-i\alpha}{1+\eta\tilde{n}+2i\left( \tilde{\Delta}-\xi\tilde{n} \right)} \right),$ | (8) |
| --- | --- |

In the above expression $\xi\tilde{n}=\frac{K_{\mathrm{nl}} n}{\kappa_{i}+\kappa_{c}}$ is the normalized nonlinear frequency shift and $\eta\tilde{n}=\frac{\gamma_{\mathrm{nl}} n}{\kappa_{i}+\kappa_{c}}$ is the normalized nonlinear two-photon loss rate, with $K_{\mathrm{nl}}\cdot n$ and $\gamma_{\mathrm{nl}}\cdot n$ being nonlinear Kerr shift and two-photon loss rate, respectively, and $\alpha$ is a small line shape asymmetry factor ($\alpha\ll1$).

The nonlinear frequency shift can lead to an asymmetric tilted Duffing response of $S_{21}(\omega)$ or a multi-solution response beyond the critical bifurcating point for $\xi<\xi_{c}$. Notably, $S_{21}(\omega)$ measurement points form a circle in an IQ plane across all parameter regimes, even for drive powers beyond the bifurcation point. Conversely, the nonlinear two-photon loss term results in a non-circular resonance curve in the IQ plane. This resonance curve takes on an approximately elliptical shape with the $S_{21}\left( \omega_{r} \right)$ and asymptotic $S_{21}\left( \omega\to\pm\infty\right)$ points located at the co-vertices, the endpoints of the minor axis.^19,20^ In the limit where $\xi\to0$ and $\eta\to0$, Eq. (8) converges to the linear generalized asymmetric Lorentzian line shape [Eq. (1)].^6^

The high-power scattering parameter data shown in Figure 5**c**,**d** is modelled by the following nonlinear fitting procedure implemented using the *lmfit* python package. Starting with initial set of fitting parameters, the photon number is computed for each frequency point using Eq. (7). These photon numbers and the remaining fitting parameters are used to compute the scattering parameter S_21_ as a function of frequency using Eq. (8). The nonlinear fitting is performed using the least square method (*leastsq*) from *scipy.optimize* package used by *lmfit*.

The nonlinear Kerr parameter ($K_{\mathrm{nl}}$) and the nonlinear two-photon loss parameter ($\gamma_{\mathrm{nl}}$) are obtained by repeating the described fitting routine for S_21_ spectra collected at higher microwave drive powers (empty markers in Figure S11**a,b**) and plotting nonlinear Kerr shift ($K_{\mathrm{nl}}\cdot n$) and two-photon loss rate ($\gamma_{\mathrm{nl}}\cdot n$) against the maximum photon number in a resonator. The nonlinear Kerr parameter and the two-photon loss parameter are finally extracted from the slope of the linear fit (Figure S11**c,d**), which are for Ta resonators exposed to diluted HF for 3 min summarized in Figure S11**e.** Surprisingly, $K_{\mathrm{nl}}$ and $\gamma_{\mathrm{nl}}$ have comparable values ranging from 0.5 to 3 kHz. This is possibly due to a common quasiparticle heating source.

Total internal quality factor combining linear intrinsic and the nonlinear loss $Q_{i}^{-1}=\frac{\kappa_{i}}{\omega_{r}}+\frac{\gamma_{\mathrm{nl}}n}{\omega_{r}}$ extracted for each S_21_ spectrum at a given power agree well with a combination of a TLS intrinsic loss model Eq. (2) and a two-photon loss from a single parameter for each resonator presented in Figure S11**e** as shown with a dashed line in Figure S11**a,b**.


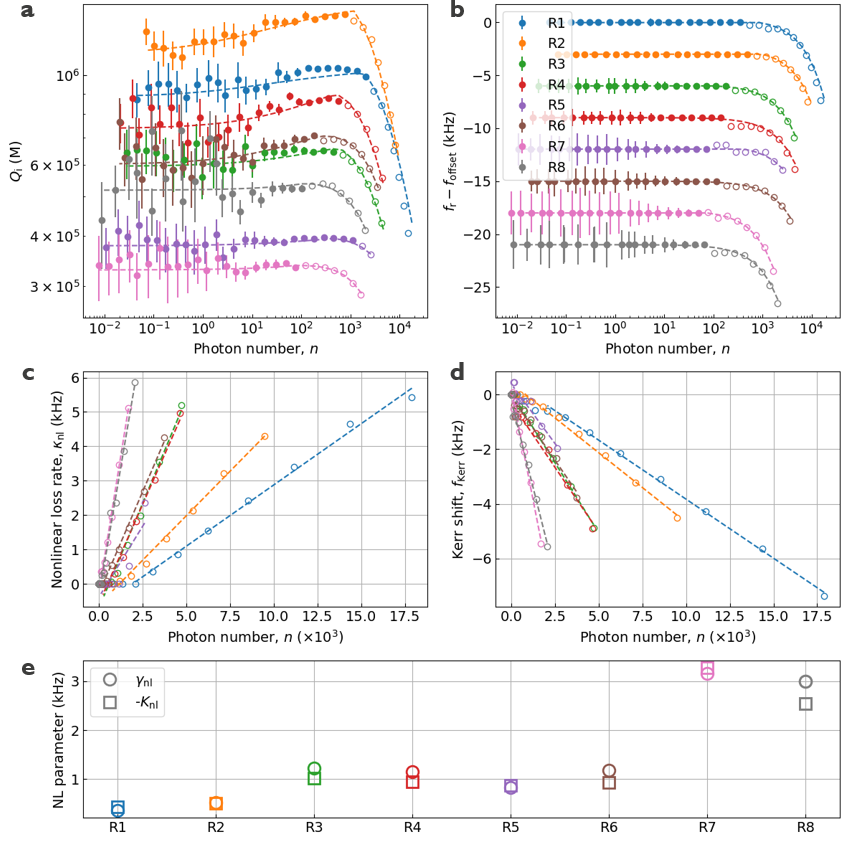


**Figure S11**: Nonlinear analysis of Q-internal and resonator frequency shift for all 8 resonators on a Ta resonator sample exposed to 3 min HF. **a** Internal Q-factor as a function of photon number in a resonator. Dashed lines correspond to total linear and nonlinear loss. **b** Resonator’s frequency shift relative to their value at the lowest photon number as a function of the photon number in a resonator. For better visibility, data for each resonator is offset by 3 kHz. Dashed lines indicate nonlinear Kerr shift. Error bars in panels a and b represent the standard errors obtained from the nonlinear fit using the lmfit python package. **c** Nonlinear loss rate as a function of photon number and **d** Kerr shift as a function of the photon number. **e** nonlinear loss (γ_nl_) and negative nonlinear Kerr (-K_nl_) parameters extracted from slopes in subpanels **c** and **d** for each resonator on a chip.


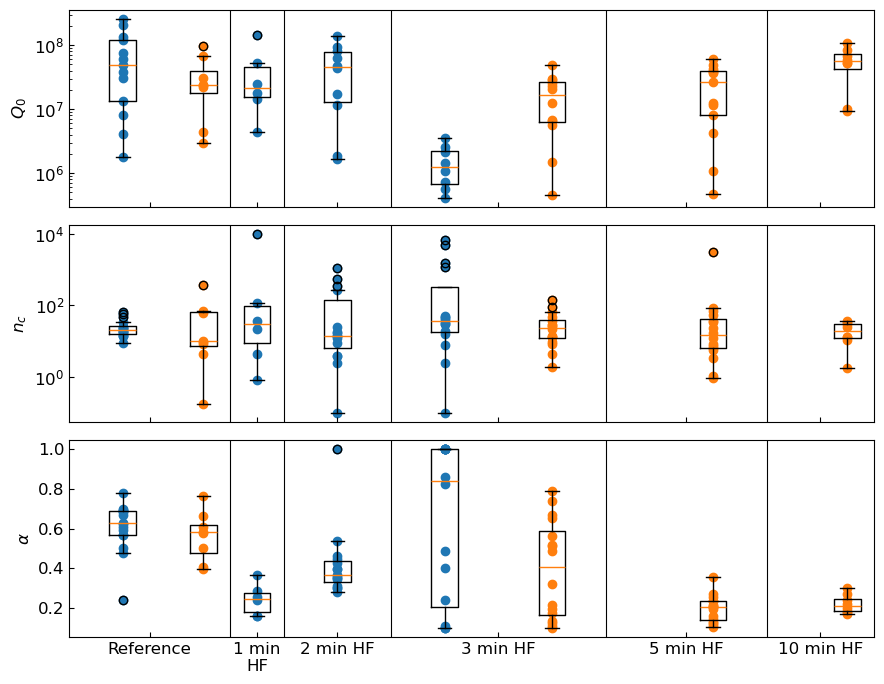


**Figure S12**: Summary of TLS model fitting parameters for different resonators with different HF treatment. High-power S_21_ measurements that exhibit nonlinear behavior were excluded from TLS model fitting Eq. (2) presented here. Parameters plotted here are: Q_0_ power-independent loss, n_c_ critical photon number, and α is the phenomenological parameter accounting for geometric effects.

# **References**

1. Verjauw, J. *et al.* Investigation of Microwave Loss Induced by Oxide Regrowth in High-Q Niobium Resonators. *Phys. Rev. Appl.* **16**, 014018 (2021).

2. Van Damme, J. *et al.* Argon milling induced decoherence mechanisms in superconducting quantum circuits. Preprint at https://doi.org/10.48550/arXiv.2302.03518 (2023).

3. Lozano, D. P. *et al.* Low-loss α-tantalum coplanar waveguide resonators on silicon wafers: fabrication, characterization and surface modification. *Mater. Quantum Technol.* **4**, 025801 (2024).

4. Rieger, D. *et al.* Fano Interference in Microwave Resonator Measurements. *Phys. Rev. Appl.* **20**, 014059 (2023).

5. Khalil, M. S., Stoutimore, M. J. A., Wellstood, F. C. & Osborn, K. D. An analysis method for asymmetric resonator transmission applied to superconducting devices. *J. Appl. Phys.* **111**, 054510 (2012).

6. Probst, S., Song, F. B., Bushev, P. A., Ustinov, A. V. & Weides, M. Efficient and robust analysis of complex scattering data under noise in microwave resonators. *Rev. Sci. Instrum.* **86**, 024706 (2015).

7. Burnett, J., Bengtsson, A., Niepce, D. & Bylander, J. Noise and loss of superconducting aluminium resonators at single photon energies. *J. Phys. Conf. Ser.* **969**, 012131 (2018).

8. Wang, H. *et al.* Improving the coherence time of superconducting coplanar resonators. *Appl. Phys. Lett.* **95**, 233508 (2009).

9. Phillips, W. A. Tunneling states in amorphous solids. *J. Low Temp. Phys.* **7**, 351–360 (1972).

10. Bruno, A. *et al.* Reducing intrinsic loss in superconducting resonators by surface treatment and deep etching of silicon substrates. *Appl. Phys. Lett.* **106**, 182601 (2015).

11. Koch, J. *et al.* Charge-insensitive qubit design derived from the Cooper pair box. *Phys. Rev. A* **76**, 042319 (2007).

12. Priebe, A., Xie, T., Bürki, G., Pethö, L. & Michler, J. The matrix effect in TOF-SIMS analysis of two-element inorganic thin films. *J. Anal. At. Spectrom.* **35**, 1156–1166 (2020).

13. McLellan, R. A. *et al.* Chemical Profiles of the Oxides on Tantalum in State of the Art Superconducting Circuits. *Adv. Sci.* **n/a**, 2300921 (2023).

14. McLellan, R. A. *et al.* Chemical Profiles of the Oxides on Tantalum in State of the Art Superconducting Circuits. *Adv. Sci.* **10**, 2300921 (2023).

15. Ranjith, P. M., Rao, M. T., Sapra, S., Suni, I. I. & Srinivasan, R. On the Anodic Dissolution of Tantalum and Niobium in Hydrofluoric Acid. *J. Electrochem. Soc.* **165**, C258–C269 (2018).

16. Chandrasekharan, R., Park, I., Masel, R. I. & Shannon, M. A. Thermal oxidation of tantalum films at various oxidation states from 300 to 700°C. *J. Appl. Phys.* **98**, 114908 (2005).

17. Zhu, M., Zhang, Z. & Miao, W. Intense photoluminescence from amorphous tantalum oxide films. *Appl. Phys. Lett.* **89**, 021915 (2006).

18. Poorgholam-Khanjari, S. *et al.* Engineering high-Q superconducting tantalum microwave coplanar waveguide resonators for compact coherent quantum circuits. Preprint at https://doi.org/10.48550/arXiv.2412.16099 (2024).

19. Yurke, B. & Buks, E. Performance of Cavity-Parametric Amplifiers, Employing Kerr Nonlinearites, in the Presence of Two-Photon Loss. *J. Light. Technol.* **24**, 5054–5066 (2006).

20. Thomas, C. N., Withington, S., Sun, Z., Skyrme, T. & Goldie, D. J. Nonlinear effects in superconducting thin film microwave resonators. *New J. Phys.* **22**, 073028 (2020).

21. Eichler, C. & Wallraff, A. Controlling the dynamic range of a Josephson parametric amplifier. *EPJ Quantum Technol.* **1**, 1–19 (2014).

22. Anferov, A. *et al.* Low-loss Millimeter-wave Resonators with an Improved Coupling Structure. Preprint at https://doi.org/10.48550/arXiv.2311.01670 (2023).

1. This line shape expression is adapted from Daniel Flanigan resonator package (<https://github.com/danielflanigan>) which is rewritten for convenient fitting with the lmfit python package. [↑](#footnote-ref-1)
